# The genome of the forest insect pest *Pissodes strobi* reveals genome expansion and evidence of a *Wolbachia* endosymbiont

**DOI:** 10.1093/g3journal/jkac038

**Published:** 2022-02-16

**Authors:** Kristina K Gagalova, Justin G A Whitehill, Luka Culibrk, Diana Lin, Véronique Lévesque-Tremblay, Christopher I Keeling, Lauren Coombe, Macaire M S Yuen, Inanç Birol, Jörg Bohlmann, Steven J M Jones

**Affiliations:** 1 Canada’s Michael Smith Genome Sciences Centre, BC Cancer, Vancouver, BC V5Z4S6, Canada; 2 Bioinformatics Graduate Program, University of British Columbia, Vancouver, BC V6T1Z4, Canada; 3 Michael Smith Laboratories, University of British Columbia, Vancouver, BC V6T1Z4, Canada; 4 Department of Forestry and Environmental Resources, North Carolina State University, Raleigh, NC 27695, USA; 5 Laurentian Forestry Centre, Canadian Forest Service, Natural Resources Canada, QC G1V4C7, Canada; 6 Département de Biochimie, De Microbiologie et de Bio-informatique, Université Laval, Laval, QC G1V0A6, Canada; 7 Department of Medical Genetics, University of British Columbia, Vancouver, BC V6T1Z4, Canada; 8 Department of Botany, University of British Columbia, Vancouver, BC V6T1Z4, Canada; 9 Department of Forest and Conservation Sciences, University of British Columbia, Vancouver, BC V6T1Z4, Canada

**Keywords:** Curculionidae, *Pissodes strobi*, spruce weevil, forest pest, genome size, transposable elements, *Wolbachia*, endosymbiont

## Abstract

The highly diverse insect family of true weevils, Curculionidae, includes many agricultural and forest pests. *Pissodes strobi*, commonly known as the spruce weevil or white pine weevil, is a major pest of spruce and pine forests in North America. *Pissodes strobi* larvae feed on the apical shoots of young trees, causing stunted growth and can destroy regenerating spruce or pine forests. Here, we describe the nuclear and mitochondrial *Pissodes strobi* genomes and their annotations, as well as the genome of an apparent *Wolbachia* endosymbiont. We report a substantial expansion of the weevil nuclear genome, relative to other Curculionidae species, possibly driven by an abundance of class II DNA transposons. The endosymbiont observed belongs to a group (supergroup A) of *Wolbachia* species that generally form parasitic relationships with their arthropod host.

## Introduction

Beetles (Coleoptera) are the largest order of insects representing more than 400,000 extant species. Amongst the beetles, Curculionidae (or, “true weevils”) are a heterogeneous taxon with more than 60,000 mostly herbivorous species that include some of the world’s most devastating forest and agricultural pests (Oberprieler *et al*. 2007; McKenna *et al*. 2009). Curculionidae evolved initially in close associations with gymnosperms, including conifers, during the Jurassic period and further diversified on angiosperms during the Cretaceous (Gunter *et al*. 2016).

*Pissodes strobi* is a highly destructive weevil pest of North American conifers requiring millions of dollars annually for screening and monitoring programs (Ebata 1991; King and Alfaro 2009). *Pissodes* *strobi* was long considered 3 distinct species due to its broad geographic and host range, but it was later recognized as a single species following multiple lines of genetic analysis (Smith and Sugden 1969; Langor and Sperling 1995, 1997; Phillips and Lanier 2000; Laffin *et al*. 2004). Common names for *P. strobi* differ in relation to geographic origin and host association (Laffin *et al*. 2004). For instance, in eastern North America, *P. strobi* is commonly known as white pine weevil as its major host in that geographic location is the eastern white pine (*Pinus strobus*). In western North America it is referred to as spruce weevil as its primary hosts there are Sitka (*Picea sitchensis*), white (P. *glauca*), Engelmann (*P. engelmannii*) spruce, as well as their hybrid (*P. glauca* × *engelmannii* × *sitchensis*). In this study, we refer to *P. strobi* as spruce weevil (Whitehill and Bohlmann 2019). 

The spruce weevil annual life cycle can be divided into 2 major phases, the exophase and endophase (Whitehill and Bohlmann 2019). During the exophase, adult weevils live on the outside of the tree and feed on its bark without causing substantial damage to the host. The endophase takes place after the female deposits its eggs into oviposition holes at the tip of the apical shoot. Larvae feeding disrupts the flow of water and nutrients and leads to apical shoot mortality. The spruce weevil is most destructive during the endophase, which continues until pupation and emergence from the tree as an adult. Damage from spruce weevil larvae results in stunted and deformed growth, and repeated infestation can result in tree death (Gara and Wood 1989).

*Wolbachia*is widespread endocellular α-proteobacteria recognized as reproductive parasite. Given its close contact with host reproductive tissues, the presence of *Wolbachia* plays an important role in host development and reproduction (Werren *et al*. 2008)*. Wolbachia* is often transmitted vertically, directly from the germ line to the offspring (Hadfield and Axton 1999) and impacts host evolution through interference of insect reproduction processes which ultimately facilitate rapid genetic selection.

To date, the genomes of more than 50 different Coleoptera species have been reported, including 8 Curculionidae species: the coffee borer beetle (*Hypothenemus hampei*) (Vega *et al*. 2015), the Argentine stem weevil (*Listronotus bonariensis*) (Harrop *et al*. 2020), red palm weevil (*Rhynchophorus ferrugineus*) (Hazzouri *et al*. 2020), oil palm pollinating weevil (*Elaeidobius kamerunicus*) (Apriyanto and Tambunan 2021), mountain pine beetle (MPB, *Dendroctonus ponderosae*) (Keeling *et al*. 2013), the easter egg weevil (*Pachyrhynchus sulphureomaculatus*) (Van Dam *et al*. 2021), the Eurasian spruce bark beetle (*Ips typographus*) (Powell *et al*. 2021), and the rice weevil (*Sitophilus oryzae*) (Parisot *et al*. 2021). Here, we report the spruce weevil nuclear and mitochondrial genomes, the genome of a putative bacterial *Wolbachia* endosymbiont, and phylogenetic comparisons to other sequenced Curculionid insect species.

## Materials and methods

### Insect rearing and DNA extraction for genome sequencing

Spruce weevils are difficult to rear from eggs in the laboratory. In this study, fourth instar larvae were isolated from the apical shoot tip of an interior spruce tree (*P. glauca* *×* *engelmanni* *×* *sitchensis*) located (50°24′N: −119°28′W) at the BC Ministry of Forests’ Kalamalka Research Station (Vernon, British Columbia, Canada) on 2013 May 6 (Whitehill *et al*. 2016b). The larvae were reared on a semiartificial diet containing a biostatic (methyl paraben) and an antifungal (sorbic acid), to reduce potential surface contaminants (Whitehill *et al*. 2016b), for 2 weeks until they entered the pupal stage. As Coleoptera larvae void their gut prior to pupation, this diet served to minimize gut-associated microbial sequences (Keeling *et al*. 2013). An individual pupa with a fresh weight of 23.3 mg was selected for genome sequencing. The insect was flash frozen in liquid N_2_ and stored at −80°C prior to DNA extraction. The sex of the pupa could not be determined prior to DNA isolation. For genome sequencing, high molecular weight (HMW) DNA was isolated by Bio S&T using a proprietary method (Montreal, QC, Canada). Initially, Bio S&T used a standard subcellular fractionation method to extract nuclei from pupal cells (proprietary information not shared). Next, HMW DNA was further purified by adding an equal volume of extraction buffer containing 3% cetyltrimethylammonium bromide (CTAB), 1% β-mercaptoethanol and 1% PVPP (polyvinylpolypyrrolidone) to extracted nuclei. DNA extracts were incubated at 65°C for 30 min followed by a chloroform extraction. DNA was precipitated and washed twice with 75% ethanol. DNA was resuspended in TE buffer. DNA integrity was confirmed by pulsed-field gel electrophoresis (PFGE). DNA weight ranged from 9 kbp to 1 Mbp with the majority >50 kbp.

### Insect rearing, isolation of nuclei, and genome size determination using flow cytometry

Second and third instar spruce weevil larvae originating from samples collected at the Kalamalka research station (see above) were isolated from the apical shoot tip of an interior spruce tree and reared on a semiartificial diet to the fourth instar as described above. Fourth instar larvae were flash frozen in liquid N_2_ and stored at −80°C. Wild-type *Drosophila* *melanogaster* adults (VWR, Cat. No. 470176-760) were used as genome size controls. DNA samples were prepared using published methods (Johnston *et al*. 2019). In brief, the head of an individual larvae was transferred into a 2 ml Dounce homogenizer containing 1 ml of Galbraith Buffer (45 mM MgCl_2_, 30 mM sodium citrate, 20 mM MOPS, and 0.1% v/v Triton X-100, pH 7.2) and kept on ice. Grinding was done with Type A pestle at a rate of 15 strokes in 10 s. Lysates were filtered through 25-µm nylon mesh (Supreme Rosin, Canada). Nuclei were centrifuged at 1,500 × *g* for 1 min at 4°C. The supernatant was removed, and the nuclei were resuspended in 650 µl of ice-cold Galbraith Buffer supplemented with 20 µg/ml of RNase A (Ambion Recombinant RNase A, Thermo Fisher). For each sample, 150 µl of nuclei suspension was transferred to a 5-ml tube (Falcon round bottom 12 mm × 75 mm, VWR) and kept as an unstained control. The remaining volume of nuclei suspension of each sample was transferred to another 5-ml tube and stained with propidium iodide (propidium iodide solution 10 mg/ml, Sigma-Aldrich) to a final concentration of 50 µg/ml. Samples were stained for at least 12 h at 4°C and protected from light before flow cytometry analysis the following day. Flow cytometry was performed at the Plateforme de cytométie du Centre de Recherche du Chu de Québec (Quebec City, Canada) on a BD SORP LSR II Flow Cytometer using BD FACS DIVA v6.1.3 to separate nuclei from debris. Data analysis was done using FlowJo v10 software (https://www.flowjo.com). We used the 2 and 4°C fluorescence from the samples (6 biological replicates), the male and female *D. melanogaster* standards (3 biological replicates for each sex) and the amount of DNA in the standards (Mulligan and Rasch 1980) to calculate the *C*-value for each sample. Genome size was converted to base pairs with the conversion factor of 978 Mbp/pg DNA.

### DNA library preparation and genome sequencing

The DNA library was prepared and sequenced at Canada’s Michael Smiths Genome Sciences Centre located at the BC Cancer Research Institute (Vancouver, BC, Canada) using methods described by Taylor *et al*. (2018). A microfluidic partitioned library was produced using the Chromium system (10x Genomics, Pleasanton, CA, USA). Gel beads-in-Emulsions (GEMs) were produced by combining DNA, master mix and partitioning oil in the 10x Genomics Chromium Controller instrument with the microfluidic Genome Chip (PN-120216; 10x Genomics). The DNA in each GEM underwent isothermic amplification as a barcode was added to each fragment. Barcoded fragments then underwent Illumina library construction (as per the Chromium Genome Reagent Kits Version 2 User Guide [PN-120229]). The resulting library was assessed for quality using the Agilent 2100 Bioanalyzer (Santa Clara, CA, USA) and a DNA 1000 assay. The median insert size was 387 bp. The library was sequenced on an Illumina HiSeqX sequencer using the paired-end protocol to produce 831 million 150-bp reads.

### Genome assembly

The National Centre for Biotechnology (NCBI) contamination screening revealed a substantial amount of sequence reads matching with *Wolbachia*. To remove the reads from the genome assembly of the spruce weevil, those were filtered using a k-mer based approach. *Wolbachia* whole genomes were accessed from NCBI GenBank as of 2019 December 16 (complete list of genomes in Supplementary Table 1) and the sequences were loaded into a Bloom filter using BioBloomTools v.2.3.2 (Chu *et al*. 2014). Reads without matches to the *Wolbachia* filter were assembled using Supernova^™^ v2.1.1 (Weisenfeld *et al*. 2017). The assembly pipeline recommends an optimal coverage of 38X to 56X, with high genome coverages being sometimes deleterious to the genome assembly contiguity. To find the optimum number of reads for the spruce weevil genome assembly, the reads were randomly and uniformly sub-sampled by Supernova –maxreads at different genome coverages (from 22X to 53X). The maximum number of total reads (53X) provided the highest N50 value (Supplementary Table 2) and was used for assembly. The draft haploid assembly was generated using the “pseudohap” argument to Supernova mkoutput. To remove heterozygosity-induced duplicated scaffolds, the Purge Haplotigs pipeline v1.0.4 (Roach *et al*. 2018) was run with parameters −l 1, −m 60, −h 200, −a 70. Tigmint v.1.1.2 (Jackman *et al*. 2018) was used to identify possible misassemblies with the parameters −s 20, −w 1,000 to break the draft assembly at regions with poor linked-read support, and scaffolded using ARKS v1.0.1 (Coombe *et al*. 2018) (−c 5, −k 30, −j 0.55, −l 0, −d 0, −e 30,000, −r 0.05). Finally, gap filling was performed iteratively using Sealer v2.2.3 (Paulino *et al*. 2015) with k-mer sizes of 90, 100, 110, and 120.

### Genome characterization from k-mers

After extracting the barcode information, the reads were trimmed to remove bases with Phred quality score ≤ 30 using Cutadapt v3.4 (Martin 2011). 21-mers were counted with KMC v3.1.1 (Kokot *et al*. 2017) with the following options: kmc –k 21 –ci 1 –cs 10,000. Smudgleplot v0.2.1 (Ranallo-Benavidez *et al*. 2020) was used to determine the lower (L) and upper (U) coverage cutoffs with the Smudgeplot.py script. The k-mers between L = 20 and U = 790 were used in smudgeplot.py hetkmers to extract the heterozygous k-mers.

The complete set of k-mer frequency histograms were computed by ntCard v1.1.0 (Mohamadi *et al*. 2017) to determine heterozygosity, repetitiveness and genome size. Frequency distributions for 21-, 23-, 25-, 27-, and 29-mers (Supplementary Fig. 1) were uploaded in GenomeScope v2.0 (Ranallo-Benavidez *et al*. 2020) assuming a diploid genome.

### Identification and annotation of protein-coding genes

We annotated the genome of the spruce weevil with supporting evidence from cDNAs and transcriptome assemblies downloaded from RefSeq NCBI (O’Leary *et al*. 2016) and Endopterygota species (taxon id: 33392), together with proteins from *D.* *melanogaster* as shown in the Supplementary Table 3. In addition, we assembled short RNAseq reads from 30 MBP libraries (SRR1702878–SRR1703019) (Keeling *et al*. 2013). Short-read RNAseq libraries were assembled with a pooled assembly approach using RNA-Bloom v1.0.0 (Nip *et al*. 2020). More details about the assembly parameters and the RNAseq samples can be found in Supplementary Table 4. Assembled transcripts were screened for contaminants and only transcripts with putative CDS (coding sequence) were used for annotation selected through EvidentialGene v2017.12.21 (Gilbert 2019). The redundancy of the transcripts was removed through CD-HIT-EST v4.8.1 (Fu *et al*. 2012) (−c 0.98 and −n 10).

The genome was annotated using MAKER2 v2.31.10 (Holt and Yandell 2011), with the annotation limited to scaffolds longer than 1 kbp, using ad hoc trained parameters for Augustus v2.5.5 (Stanke *et al*. 2006), SNAP 2006-07-28 (Korf 2004) and GeneMark v2.3c (Lomsadze *et al*. 2005) gene predictors. Augustus retraining was performed with BUSCO v3.1 (–long option) (Waterhouse *et al*. 2018) using the Endopterygota core gene set in odb9. SNAP was trained with high-quality gene models generated by a preliminary run of MAKER, selected by the minimum eAED score of 0.5 and by the fraction of splice sites predicted by SNAP of at least 0.5. GeneMark was self-trained as GeneMark-ES with an unsupervised procedure where the algorithm parameterization was solved automatically. Repetitive elements were identified with the repeat library described below, and used as a customized library during the annotation process. EnTAP v0.92 (Hart *et al*. 2020) functional annotation package was used to remove unlikely gene annotations and select a high confidence gene set better suited for biological analyses. EnTAP was provided with 2 databases; NCBI RefSeq 99 and Swiss-Prot/TrEMBL (Uniprot 2021) downloaded in April 2020. Three criteria were considered to generate the high confidence gene set: (1) all splice sites in the predicted genes must be supported by a canonical splicing motif (GT–AG, GC–AG, AT–AC at the donor and acceptor splice sites); (2) intron sequence longer than 9 bp; and (3) the protein sequence contains both START and STOP codons.

### Quality assessment of genome assemblies and genome annotations

The genome assembly was assessed by aligning the filtered 10x Chromium linked-reads (*Wolbachia* reads filtered out) to the assembled genome using the Sequence Quality Assessment Tool (SQUAT) (Yang *et al*. 2019), which evaluates the sequence assembly through the percentage of Poorly Mapped reads (PM%). The gene space completeness was assessed with BUSCO v5.2.1 (−m genome option) and the Endopterygota library in odb10 (*n* = 2,124). The genome was also assessed with BUSCO v4.1.4 odb10 and BUSCO v3.1 odb9 (n = 2,442) and reported in Supplementary Fig. 2. The annotated proteins were assessed with BUSCO v5.2.1 (−m protein option) and the Endopterygota core dataset odb10.

### Phylogenetic comparisons

The assembled spruce weevil genome was compared to a set of selected Coleopteran species as shown in Supplementary Table 5. Phylogenetic relationships were inferred through complete and single-copy orthologs annotated with BUSCO v5.2.1 and the Endopterygota lineage core gene set odb10. Protein sequences in each ortholog group were aligned with Mafft v7.453 with –auto option (Nakamura *et al*. 2018), then RAxML v8.2.12 (Stamatakis 2014) was used to build gene trees (PROTGAMMAAUTO and 100 bootstraps). Fragmentary genes (i.e. sequences with gaps in more than 67% of the sequence sites in the multiple-sequence alignment) were excluded from the downstream analysis. The species tree was estimated with ASTRAL v5.6.3 (Zhang *et al*. 2018) and results were evaluated with the relative frequency analysis in DiscoVista v1.0 (Sayyari *et al*. 2018). To determine the number of conflicting genes, the gene trees were rerooted on the red flour beetle (*Tribolium* *castaneum*) and compared to the species tree. The estimated concordance was evaluated with phyparts (Smith *et al*. 2015) and the percentage of concordance for each tree node was downloaded and used in the species phylogeny.

### Annotation and quantification of repeat elements

The repeat annotation and quantification approach was applied to both the spruce weevil and MBP genomes using the same methodology to allow an unbiased comparison. The repeat elements were predicted separately on each genome with a combination of transposable elements (TEs) Annotator pipeline for the long terminal repeat (LTR), terminal inverted repeat (TIR), and Helitron repeats (EDTA v1.8.4) (Ou *et al*. 2019) and RepeatModeller v2.0.0 for the de novo identification of genomic repeat elements (Flynn *et al*. 2020). These elements were combined with RepBase v22.08 (Bao *et al*. 2015) to yield each final custom library of repeat elements. The Kimura 2-parameter sequence divergence was estimated within each family using the calcDivergenceFromAlign.pl script from RepeatMasker v4.0.9 (Tarailo‐Graovac and Chen 2009). For additional repeat quantification, RepeatExplorer (Novák *et al*. 2013) was used with the unassembled reads from *s*pruce weevil and, for comparison, existing sequences for the MPB (SRR546176–SRR546191). The complete set of reads was trimmed with Cutadapt v3.4 (Martin 2011), bases with quality score ≤ 30 were removed (–minimum-length 120, –maximum-length 120, –l 120, –q 30), and unpaired reads were removed. RepeatExplorer was run locally as a comparative analysis between the 2 species in paired-end mode, with default minimal overlap of 66 bp. Only read clusters with at least 0.01% of the input reads were annotated with the repeat library generated from the genome assembly. The classification of satellite repeats was performed through the TAREAN database (Novák *et al*. 2017). The clusters were manually annotated with the main repeat classes: a cluster was assigned if more than 50% of the reads are uniquely assigned to a repeat family. Clusters with reads that do not reach the majority in any class are labeled as “mixture” repeats.

### Mitochondrial genome assembly and annotation

Reads were randomly sub-sampled to reach the optimal coverage for organellar assembly (46, 25, 12, 6, 3, and 1.5 million of read-pairs), as previously described (Lin *et al*. 2019). Each subset of reads was assembled using ABySS v2.1.0 (Jackman *et al*. 2017) with varying values of k and kc (k: 48, 64, 80, 96, 112; kc: 3, 4). The 2 largest scaffolds identified by similarity to the spruce weevil reference mitochondrial genome (MH404102.1) were used to recruit the reads for the final assembly through alignment with bwa mem v0.1.7 (Li and Durbin 2010).

The recruited reads were assembled with Unicycler v0.4.7 (Wick *et al*. 2017) with coverage 62X, 96X, and 128X. The assembly graphs were visualized and inspected with Bandage v0.8.1 (Wick *et al*. 2015). The assembly at 128X coverage was selected for downstream assembly and circularization. The 2 largest scaffolds from the selected assembly were combined together with an estimated gap size of 535 N. The assembly was circularized according to the reference, introducing a 10-N gap. The resulting circularization and scaffolding gaps were filled with Sealer v2.1.0 and the genome was polished with Pilon v1.22 –fix all (Walker *et al*. 2014) for fixing single nucleotide polymorphisms (SNPs), indels, misassemblies, and additionally filling scaffolding gaps. The final assembly was manually polished to reduce the remaining scaffolding and circularization gaps respectively to 530 and 8 N. The short circularization gap remains unresolved likely due to the low complexity AT-rich region.

The assembly was annotated with MITOS 2 v2017-09-02 (Bernt *et al*. 2013) using the invertebrate genetic code and RefSeq 63 Metazoa. The functional annotation of the genes matches the human mitochondrial annotation (NC_012920.1). Four genes required manual annotation (COX1, ND3, ND5, and ND4) at their terminal stop codons (TAA). All expected genes were annotated despite the unresolved gap in the assembly.

### *Wolbachia* genome assembly and annotation

The selected *Wolbachia* reads identified with the strategy described above and corresponding to 2.6 million of read-pairs (∼0.6% from the sequenced reads) were assembled with ABySS v2.2.3, using a range of k values (34, 44, 54, 60, 68, 70, 72, 74, 76, 78, 80, 82, 84, and 86) and read sub-samples at 50X, 100X, 200X, and 300X coverages. The assembly with the highest scaffold N50 and the highest genome reconstruction size (Supplementary Table 6), corresponding to k = 68 and the maximum number of reads (375X), was selected for further analysis. Tigmint v1.1.2 and ARKS v1.0.1 were used in an attempt to improve the draft assembly to no apparent benefit. The draft from ABySS was subsequently gap filled using Sealer v2.2.3 as described previously for the spruce weevil genome.

The genome was annotated with NCBI Prokaryotic Genome Annotation Pipeline (PGAP) v5.2. The annotated genes were checked for uniqueness with BLASTP all-vs.-all alignments; proteins were considered as multiple copies if the alignment had an amino acid sequence identity over 95% and alignment coverage over 90%.

### Phylogenetic comparisons and supergroup classification of the putative *Wolbachia* endosymbiont

The putative *Wolbachia* endosymbiont from spruce weevil was compared against well-characterized *Wolbachia* spp. associated with insect hosts and described by a supergroup class. The phylogenetic comparison consists of 28 *Wolbachia* genomes retrieved from NCBI GenBank and four outgrouping *Rickettsias* species, as listed in Supplementary Table 7. The multispecies *Wolbachia* phylogeny was built using the single-copy genes from the proteobacteria lineage core dataset, predicted by BUSCO v3.1 (–long option). The annotated proteins were aligned and clustered with Mafft v7.453 with –auto option and RAxML v8.2.12, respectively. The final *Wolbachia* phylogeny tree was built with ASTRAL v5.6.3 and scored by quartet supporting values calculated with the −q option.

## Results and discussion

### Genome assembly

We sequenced the draft genome of the spruce weevil at 53X coverage, using short 10x Chromium linked-reads, and assembled the reads to obtain a draft genome with 1.83 Gbp reconstructed genome size (Table 1) and a scaffold NG50 of 87.7 kbp. A noted challenge in reconstructing this genome was the high heterozygosity rate of the spruce weevil genome sampled, often impeding haploid genome resolution and causing over-assembly. To compensate, we used Purge Haplotigs to remove 80,527 heterozygosity-induced duplicated scaffolds, equivalent to 0.4 Gbp. The resulting assembly was corrected for potential misassemblies with Tigmint and rescaffolded with ARKS, and its 9,207 scaffold gaps were closed with Sealer.

**Table 1.** Assembly statistics for each assembly step.

	Supernova	Purge Haplotigs	Tigmint	ARKS	Sealer
No. scaffolds	163,521	82,994	84,653	82,897	**82,896**
Longest scaffold (kbp)	2374.58	2374.58	2139.77	2209.50	**2210.97**
Contigs NG50	4,794	4,798	5,332	4,459	**4,451**
Scaffold NG50	79,498	79,343	74,900	87,586	**87,740**
Reconstruction size (Gbp)	2.23	1.83	1.83	1.83	**1.83**
BUSCO complete (%)	79.8	79.2	79.3	79.6	**79.6**
BUSCO duplicated (%)	12.2	8.2	8.6	8.6	**8.6**

The final assembly statistics are highlighted. The values are calculated for scaffolds longer than 1 kbp. The estimated genome size for computing NG50 is 1.83 Gbp. The Endopterygota BUSCO core gene set (n = 2,124) was used to estimate the gene completeness.

The assembly quality was evaluated by mapping the reads back to the genome to flag potentially misassembled genomic regions. The mapping quality metric generated by SQUAT and bwa mem reports only 6.5% of the reads as poorly mapped (PM%), thus passing the assembly assessment successfully according to the software default settings (Supplementary Figs. 3 and 4).

In total, 79.6% (1,691) of the Endopterygota core gene sets were found to be BUSCO “Complete.” When “Fragmented” genes were included, the number detected rose to 93.3% (1,983) BUSCO core gene set (see Supplementary Fig. 5). After Purge Haplotigs, we observed a decrease in the number of duplicated genes from 12.2% to 8.2%, which likely indicated a successful removal of duplicated scaffolds from the genome assembly.

### Genome annotation

The MAKER2 gene annotation pipeline identified 19,484 genes that had a sequence similarity with known proteins (Table 2). The majority of those (18,106) were assigned to a gene ontology term that best described their function (Supplementary Fig. 6) and the MAKER annotation pipeline predicts 58.6% (1,244) of the total BUSCO genes as “Complete” (“Single-copy” and “Duplicated”). Annotated genes had high similarities to sequences from other Curculionidae species present in the databases. About 42% of all annotated genes had a homologous match with either rice weevil or MPB sequences (Supplementary Fig. 7). The quality selection criteria used to identify a high confidence set of genes reduced the number of genes to 11,382 annotating 42.9% (912) of the total BUSCO genes “Complete” (Supplementary Fig. 8). The lower percentage of BUSCO “Complete” genes annotated by the MAKER compared to the genome BUSCO “Complete” possibly represents a limitation of the MAKER pipeline. This discrepancy likely depends on the MAKER gene model training that misses the annotation of some gene families. Altogether, the total coding bases of the annotated genes covered 1.1% and 0.7% of the total genome for the total number of genes and the high confidence gene set, respectively.

**Table 2.** Annotated genes and transcripts statistics for all and the high confidence gene datasets.

	No. genes	No. transcripts	Total coding bases (Mbp)	BUSCO complete (%)	BUSCO duplicated (%)
Total annotated	19,484	19,532	22.69 (1.1%)	58.5	6.1
High confidence	11,382	11,405	14.51 (0.7%)	42.9	3.7

The total annotated genes are shown as the total coding bases and their corresponding percent from the genome. The BUSCO completeness (total BUSCO “Complete”) was used to estimate the annotation quality.

### Nuclear genome characteristics based on in silico analyses

The short-read data were used to assess spruce weevil genome features, such as ploidy structure, heterozygosity, repeats, and genome size. The final result of Smudgeplot supported a diploid genome (Supplementary Fig. 9) with the most abundant heterozygous k-mer pair (64%) being AB. Using the diploid k-mers modeling in GenomeScope, the estimated average genome size was 1.76 Gbp over the k-mers range of 21–29 (Supplementary Table 8). The estimate is confirmed by the Supernova genome assembler, which calculated a genome size of 1.78 Gbp. The genome is highly heterozygous, with the first peak in the k-mer frequency distribution being higher than the second peak. GenomeScope estimates an average of 2.56 heterozygous bases every 100 bases.

### Nuclear genome size estimation by flow cytometry

The spruce weevil genome size was estimated based on the amount of DNA contained within the haploid nucleus (*C*-value) using flow cytometry*.* Fruit fly (*D.* *melanogaster*) was the external reference genome used as a positive control. The estimated haploid genome size of spruce weevil was 2.07 ± 0.05 pg (mean ± SD), or 2.02 ± 0.05 Gbp. Flow cytometry analysis of spruce weevil DNA provides further support to the in silico estimates for the reconstructed genome size. The range previously reported for weevils (Curculionidae) is currently limited to 8 species which vary from 0.20 to 3.25 Gbp (http://www.genomesize.com).

### Phylogenomics analysis

**Fig. 1.**
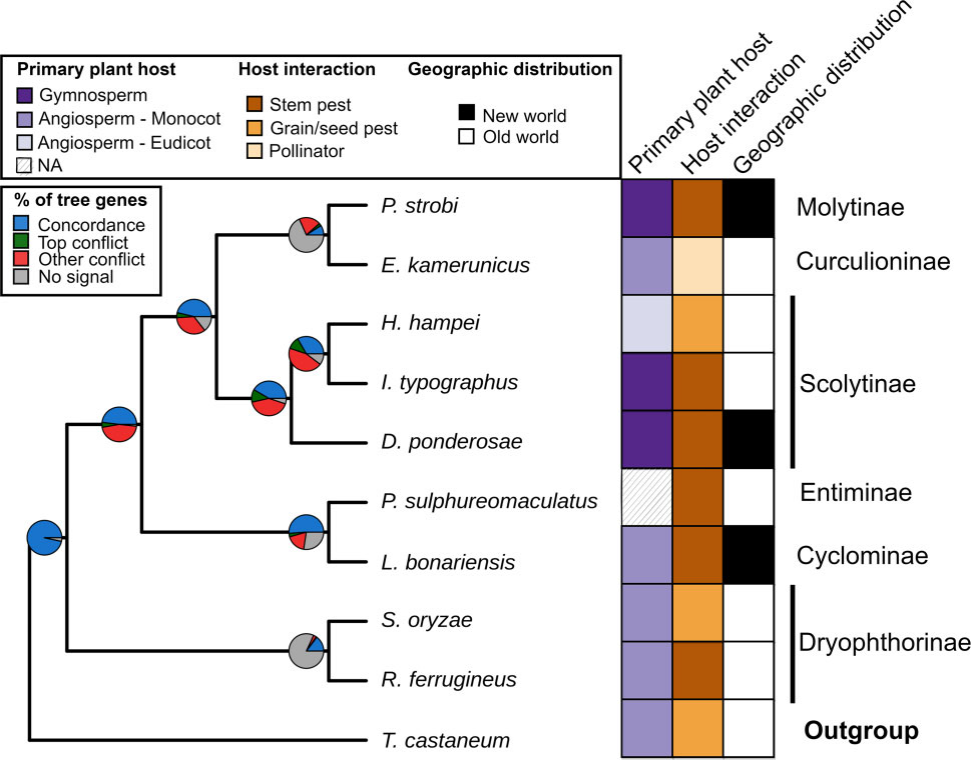
Phylogenomic species tree reconstructed from BUSCO annotated genes. The supporting genes at each internal node are shown in blue (“Concordance”). Green and red are the number of genes conflicting with the species tree and supporting respectively one main alternative (“Top conflict”) or other alternatives (“Other conflicts”). Gray is the number of missing genes for the species tree. The lpp is 100% for all the internal nodes. The block graph classifies the Curculionidae based on their plant host, host interaction and geographic origin; the subfamily is shown for each species.

We applied a broad phylogenomics analysis based on BUSCO single-copy gene annotations, comparing 9 species of Curculionidae and red flour beetle in the out group as model species for Coleopterans. We identified 63% of the orthologous genes reconstructed in at least 8 out of 10 species, with only 3% of poorly overlapping orthologs (reconstructed in less than 6 species; Supplementary Fig. 10). Only a small fraction of the genes (43 from 16,461 total genes) was labeled as fragmentary and excluded from the phylogeny due to a large fraction of gaps in the multiple sequence alignments suggesting divergence from the other annotated sequences. Given the large overlap of BUSCO annotated genes, we were able to use a total of 2,080 BUSCO orthologous genes for phylogeny inference. The degree of concordance was added as pie charts to the species tree in Fig. 1 to summarize the genes supporting contribution under the multispecies coalescent model. The resulting species tree had strong support from the underlying gene topology with a local posterior probability (lpp) of 100% and 69% of the quartet trees represented in the species tree. The subfamily grouping of the Curculionidae was correctly represented, with the Scolytinae or bark beetles (MPB, Eurasian spruce bark beetle and coffee borer beetle) and the Dryophthorinae or pantropical weevils (rice weevil and red palm weevil) clustering closely. The spruce weevil from the Molytinae subfamily groups together with oil palm pollinating weevil which is taxonomically classified as flower weevil (Curculioninae). Despite their proximity in the species phylogeny, the number of genes with missing signal remains dominant. The number of genes with conflicting topology remains high when compared to the genes having a concordant topology (Supplementary Figs. 11–13; 446 and 187), thus supporting the different subfamily classification of the spruce weevil and oil palm pollinating weevil*.* Notably, together with the high uncertainty of spruce weevil and oil palm pollinating weevil, the relative quartet frequency calculated by ASTRAL and DiscoVista shows uncertainty also for the node between Eurasian spruce bark beetle and coffee borer beetle (Supplementary Fig. 12).

The phylogeny of Curculionidae has been previously reconstructed using a limited number of molecular markers, applying at most >500 single-copy orthologs as in Shin *et al*. (2018). Given the growing number of Curculionidae genomes for comparison, we employed 2,080 single-copy orthologs from the whole nuclear genome that is, to our knowledge, the largest number of gene markers used for Curculionidae phylogeny. We compared the spruce weevil to 8 Curculionid species, representative of 5 different subfamilies. The spruce weevil is the unique representative of the Molytinae subgroup in our analysis and in the phylogeny showed similarity to a member of the Curculioninae, oil palm pollinating weevil. The comparison included 2 additional species that infest gymnosperm trees. Despite differences in host preference, spruce weevil shares a higher genomic similarity to a “pollinator” weevil that feeds on angiosperms. These results are striking as spruce weevil is further removed from the bark beetles subfamily (some of the most destructive forest pests known) than a mutualistic pollinator species. The results suggest that speciation and subfamily differentiation is not strictly connected to the host plant. Host preference also differs within the Scolytinae subfamily. The major host of bark beetle coffee borer beetle is an angiosperm, while MPB and the Eurasian spruce bark beetle are some of the most destructive pests of conifer forests in the world.

### Expansion of repeat families and genome size

**Fig. 2.**
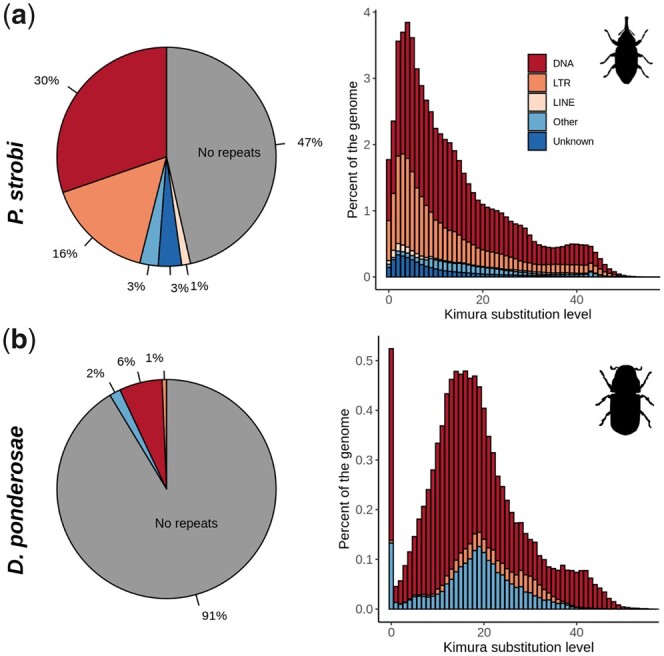
Repeat composition of a) spruce weevil and b) MPB genomes. Repeats are shown as a percentage from the total genome (left) and repeat landscape plots (right). Repeats are grouped into 5 major classes. The Kimura substitution level estimates the repeat divergence calculated respect to a consensus repeat library. Higher values of substitution indicate a more diverging sequence. “Other” repeats include Mavericks, Penelope, DIRs, and Helitrons.

The spruce weevil genome was annotated for both de novo and motif-based repeats, resulting in TE repeat library that marked a high fraction of the genome as repetitive (Fig. 2). The spruce weevil genome was highly enriched for repeats, with more than half the genome (53.53%) annotated as repetitive (Fig. 2a and Supplementary Table 9). Conversely, the repeat content of the closest Curculionidae forest pest, MPB, contains only 8.65% total genomic repeats (Fig. 2b and Supplementary Table 9), which correlates with its smaller genome size of 0.204 Gbp as reported by MPB genome size study and its reconstructed genome size (Gregory *et al*. 2013; Keeling *et al*. 2013). The most abundant class of repeats are class II DNA transposon repeats, which cover about 30% of the genome, and the DTA/hDA repeat class, covering 15% of the total genome (Supplementary Table 9). The class I TE repeats covered about 16% of the total genome, with Gypsy being the most abundant class at 8%. The percentage of genomic repeats represents draft estimate which are valuable to compare given the complete reconstruction size of the two genome assemblies, even if the genome of the MPB was sequenced with an older short-read technology. It is possible that the two assemblies may have higher repeat content than the one presented in here. This is because repeat sequences are notoriously difficult to assemble with short reads which will result in short scaffolds that are not included in the final assembly.

We further quantified repeat abundance from the unassembled genomic reads using RepeatExplorer. The pipeline randomly selected a set of 1,573,630 read-pairs. Reads recognized as repeats were arranged in 94,640 clusters with ∼52% of the sequences identified as repetitive (Supplementary Fig. 14). The top 335 clusters covered 29% of the selected reads. The total read counts of those 335 clusters are plotted in Supplementary Fig. 15 and show a similar genome repeats content as in Fig. 2, a and b, with a higher count of DNA and LTR repeats in spruce weevil.

Using the genome-derived repeats library, we analyzed the sequence divergence measured by Kimura distance (rate of transitions and transversions) within the two species and found that the distance of the two genomes had a significantly different repeat landscape profile. The spruce weevil (Fig. 2a) showed a large spike of proliferation for all of the DNA, LTR, and Unknown type of repeats between the Kimura distance of 3% and 6%. MPB did not have a similar peak but instead had a diverse profile with two major peaks (Fig. 2b), one at the most recent peak and the other in the range distance between 14% and 20%. Both the species showed a similar pattern of repeat proliferation around 40% Kimura distance, which indicates a common repeat evolution pattern before their speciation.

Comparisons to other published Curculionidae species highlighted the relatively large genome of spruce weevil (e.g. the Easter egg weevil, *P. sulphureomaculatus*, has a comparable genome size of ∼2 Gbp). Agreement between experimental and in silico analyses supports a nuclear spruce weevil genome size of ∼2 Gbp. The weevil genome was annotated with a large abundance of TE, which are positively correlated with genome size in arthropod species (Petersen *et al*. 2019). The most abundant class of repeats in the spruce weevil genome are DNA transposons, class II type of repeats (i.e. cut-and-paste). Class II TE are known to contribute to genome size increases and may indicate a complex replicative transposition based on homology-dependent repair (Skipper *et al*. 2013). The most abundant DNA transposons in the spruce weevil genome are the *hAT* (DTA) and *Mutator* (DTM) families that are ubiquitous in eukaryotes. The two families are found in large copies in the genome, each spanning several kbp in length (3–12 kbp).

Another interesting aspect of the spruce weevil genome relates to the timing of TE expansion and the identification of active classes. Figure 2b shows repeat amplifications and extinction, phenomena represented by peaks and valleys in the graph. The Kimura repeats landscape highlights a recent expansion event that could have dramatically reshaped the genome structure. Specifically, a comparison between spruce weevil and MPB (Keeling *et al*. 2013) revealed high variability in genome composition. The findings suggest an expansion of lineage-specific repeats but also different mechanisms of TE elimination that may have acted differently in these two species. Most of the TE copies in the spruce weevil show a divergence below 10%, suggesting high repeat replication and turnover rates in recent time when compared to the MPB. Both DNA and LTR type of repeats show a wide spectrum of divergence estimates in the spruce weevil, with a significant peak around 5%. These two types of repeats show a synchronous expansion, which indicates a related turn-over event. Finally, the “Unknown” type of TEs that covers about 6% of the total genome has a steady expansion also culminating with a peak around 5%.

### Mitochondrial genome

The final mitochondrial assembly of the spruce weevil consisted of one unique scaffold of 16,222 bp. The final scaffold had a GC content of 24% and a final gap size of 538 Ns that did not overlap with the annotated genes. In total, 3 different assembly graphs were produced representing different coverages to inspect the assembly results (Supplementary Figs. 16–18). The final mitochondrial genome assembly (Supplementary Fig. 18) was given a circular structure according to the reference. A total of 37 genes were annotated including: 13 protein-coding, 22 tRNA-coding, and 2 rRNA-coding genes. The same 37 genes are found in the mitochondrial sequence of *Hylobitelus xiaoi* (NC_022680.1), the closest annotated relative with an available mitochondrial genome to spruce weevil.

### *Wolbachia* genome assembly

The putative *Wolbachia* endosymbiont genome consists of 247 scaffolds longer than 1 kbp. It has 1,608,243 bp and an average genomic GC content of 40%. A total of 1,588 complete genes were annotated including: 1,550 protein-coding, 4 rRNA-coding and 34 tRNA-coding genes. We investigated the uniqueness of the protein-coding genes annotated in *Wolbachia* and we did not detect significant sequence similarity between any pair of bacterial proteins.

It is possible that the quality of the draft *Wolbachia* genome assembly is impacted by host genome integration events. In the case of genome integration, the genomic regions of the *Wolbachia* endosymbiont would be flanked by genomic regions from the host genome, which would not allow for contiguous assembly. However, given that the contiguity of the *Wolbachia* genome is spread across 247 scaffolds with a reconstruction size of 1.60 Mb, current evidence does not support integration of the endosymbiont genome into the host.

### *Wolbachia* supergroup phylogeny

**Fig. 3.**
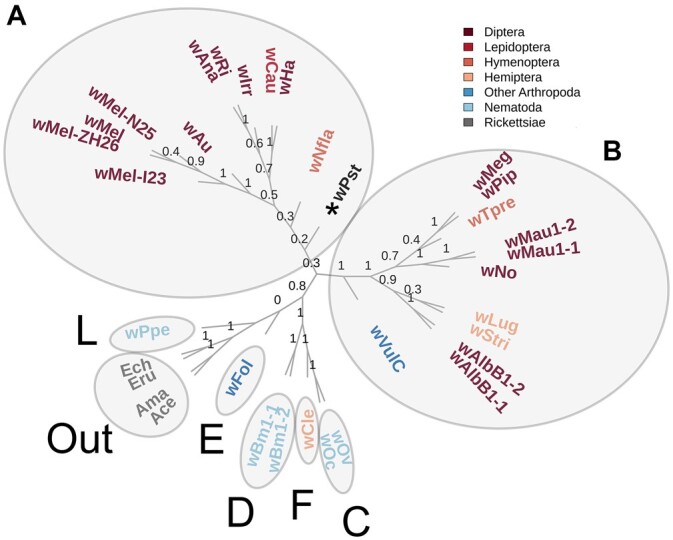
Phylogenomics tree of *Wolbachia*, reconstructed from BUSCO single-copy proteobacterial genes. The samples are colored based on their order classification as for insects, nematodes, and other Rickettsiae species. The internal branches show the lpp based on the supporting genes. wPst (the *Wolbachia* endosymbiont in the spruce weevil host) is marked with an asterisk and it is the only one bacterial endosymbiont from a Coleopteran host species.

A phylogenetic tree comprising well-characterized *Wolbachia* spp. was constructed to classify the newly-assembled genome of the putative spruce weevil endosymbiont. A total of 193 proteobacteria BUSCO single-copy genes were used to build the phylogenetic tree since they were found in most of the annotated species (115 genes were reconstructed in all the species and 177 in more than 80%). The phylogenetic tree grouped the samples belonging to supergroup A together in a common cluster (Fig. 3), as it does for supergroup B. The supergroups C, D, E, F, and L and the 2 Rickettsiae outgroups (Ama, Ace; Ech, Eru) were grouped together in the third major cluster, showing a higher heterogeneity. *Wolbachia* associated with a *D. melanogaster* host closely grouped together, while the spruce weevil associated putative *Wolbachia* endosymbiont (wPst) grouped with samples belonging to supergroup A. The spruce weevil endosymbiont wPst shared a higher sequence similarity with the supergroup A cluster but also showed a diverging position when compared to the other species within that supergroup. The other samples in supergroup A grouped tightly when isolated from the *D. melanogaster* host (wMel), while the samples on the opposite branch were isolated from *D. simulans*, *D. ananassae*, and *C. sasakii* (wHa, wRi, wRau; wAna and wCau, respectively). These findings support that *Wolbachia* can share high levels of similarity despite being derived from very different host species.

*Wolbachia* infections can be positive (Hosokawa *et al*. 2010), but are generally considered a reproductive parasite that can negatively impact host insect fitness (Werren *et al*. 2008). The quality of a conifer host can significantly impact the reproductive fitness (i.e. ovary development) of adult spruce weevil females prior to oviposition. However, an ecological mechanism has not yet been described (Robert and Bohlmann 2010).

The presence of bacterial endosymbionts has been extensively described in rice weevil where specialized host cells called bacteriocytes have been found to contain both specialized endosymbionts and *Wolbachia* (Heddi *et al*. 1999). Detailed studies of bacteriocyte tissues of spruce weevil at the cellular and subcellular level have not yet performed. However, the structures that house bacteriocytes (i.e. bacteriomes) have been previously documented and described in spruce weevil (Whitehill *et al*. 2016b). Differences in morphology of these structures following development on resistant and susceptible host genotypes were also observed (Whitehill et al. 2016a, 2019). Also, a role for the *Wolbachia* endosymbiont could have been impacting previous observations regarding the interaction between spruce weevil and adult female weevil fecundity (Robert and Bohlmann 2010). Prolonged interactions between a host and its *Wolbachia* endosymbiont can result in the evolution of a bacteriocyte-associated obligate mutualism (Hosokawa *et al*. 2010). For instance, the diversity of species in supergroup A indicate that closely related *Wolbachia* strains are found in a diverse array of host species. Further studies and targeted experiments can give more information about the specific mechanisms involved in this process.

## Summary and conclusions 

Due to difficulties in maintaining laboratory colonies of spruce weevil we chose to sequence a wild-collected insect as has been the strategy for other Curculionid species (Keeling *et al*. 2013). The sequenced individual was reared from an egg on a semiartificial diet to reduce potential contaminants. The nuclear and mitochondrial genome assemblies are reconstructed from a single pupa, sequenced with 10x Chromium linked-reads technology at 53X coverage. We have assembled the nearly complete mitochondrial genome of the spruce weevil, completed with annotation refined by manual curation. Interspersed among the raw DNA reads was a high percentage of sequences that matched to a bacterial species most closely resembling *Wolbachia*. The nearly complete *Wolbachia* genome sequence was assembled de novo and the annotation resulted in a large number of complete genes.

Compared to existing genomic resources for closely related species, the spruce weevil genome is complex, highly heterozygous, repeat-rich, and significantly larger than other sequenced forest pests, such as MPB. For the spruce weevil nuclear and mitochondrial genome assemblies we used 10x Chromium linked-reads to enable the phasing of long-range information that groups together genomics fragments. We have used a linked-reads assembly strategy to resolve heterozygous parental alleles which still remains a challenge in the assembly of nonmodel organisms. Our analyses of the spruce weevil genome size expansion revealed unique aspects of TE. However, questions relating to the biological underpinnings of genome size expansion in spruce weevil still remain. Specifically, it is unclear how geographically separated populations of spruce weevil maintain gene flow amongst groups. Given the wide host range of spruce weevil coupled to its large geographic footprint, a comprehensive analysis of individuals adapted to different hosts and regions could reveal clues related to the size expansion within this Curculionid species.

## Data availability

The nuclear genome reads are available on SRA, SRX7270456; the sequence data and assembly are available in NCBI GenBank under the accession JAEQML000000000, BioProject PRJNA588625. The mitochondrial genome assembly was submitted to NCBI GenBank under the accession MW452482; the *Wolbachia* genome assembly is under GCA_019097885. The gene annotations and repeat library are available on the BCGSC site: https://www.bcgsc.ca/downloads/supplementary/WhitePineWeevil.

Supplemental material is available at *G3* online.

## Supplementary Material

jkac038_Supplemental_Figures

jkac038_Table_S1

jkac038_Table_S2

jkac038_Table_S3

jkac038_Table_S4

jkac038_Table_S5

jkac038_Table_S6

jkac038_Table_S7

jkac038_Table_S8

jkac038_Table_S9
