# Supplementary material for: The genome of the forest insect pest Pissodes strobi reveals genome expansion and evidence of a Wolbachia endosymbiont
Source: G3 (Bethesda). 2022 Feb 16;12(4):jkac038. doi: 10.1093/g3journal/jkac038 (PMC8982425; doi:10.1093/g3journal/jkac038)
Supplement: jkac038_Supplemental_Figures [file jkac038_supplemental_figures.pdf]

Supplementary Figures

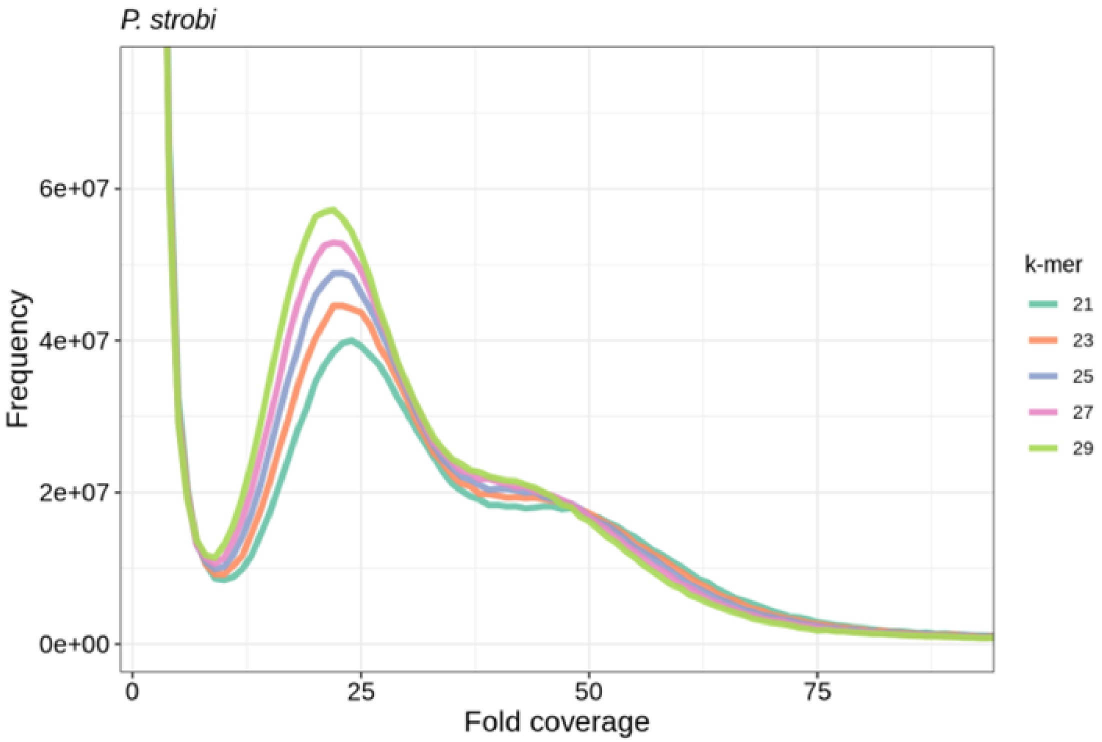

Fig. S1 Genome characteristics from k-mers: k-mer profiles calculated by ntCard and used to characterize the genome of *P. strobi*. The plot shows the frequency of kmers on the y-axis and the fold coverage on the x-axis for the five chosen k-mer sizes. The curve shows the erroneous k-mers in the peak between 0 and 3 fold coverage, the homozygous k-mer (common in both parental alleles) as the maximum peak in the histogram and the last peak (around 50) for the heterozygous k-mers.

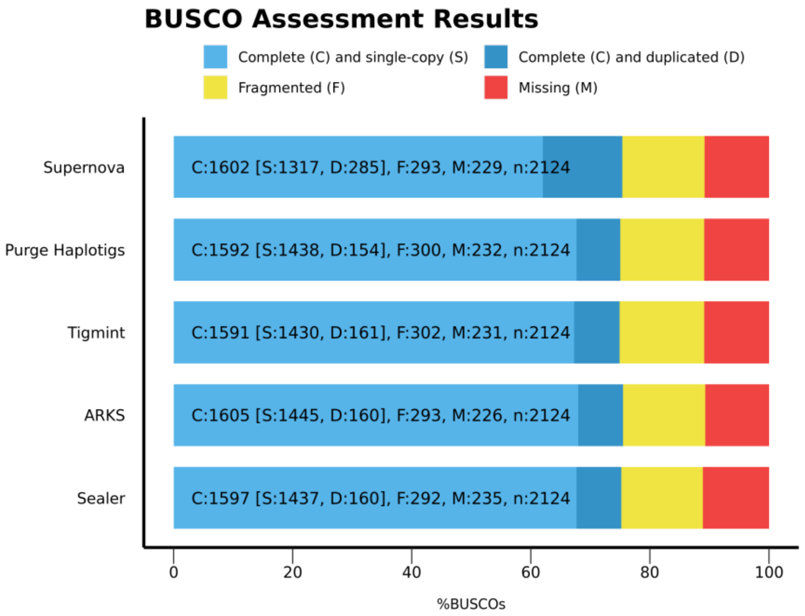

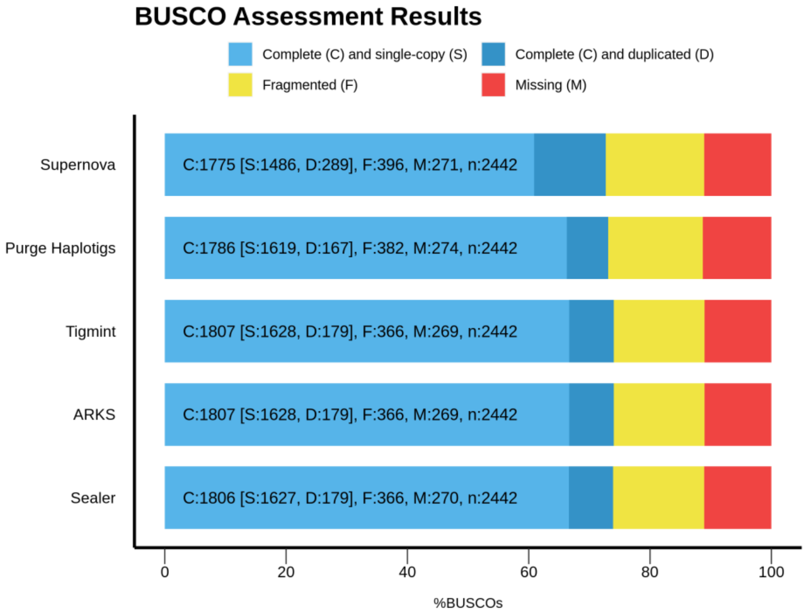

Fig. S2 Quality assessment of genome annotation: BUSCO assessment results for each stage of the genome assembly – bottom is the last stage of the assembly. The plot reports the percentage of "(C)omplete – (S)ingle copy", "Complete (D)uplicated, "(F)ragmented" and "(M)issing" Endopterygota core gene set for BUSCO v4.1.4 odb10 (top) and BUSCO v3.1 odb9 (bottom)

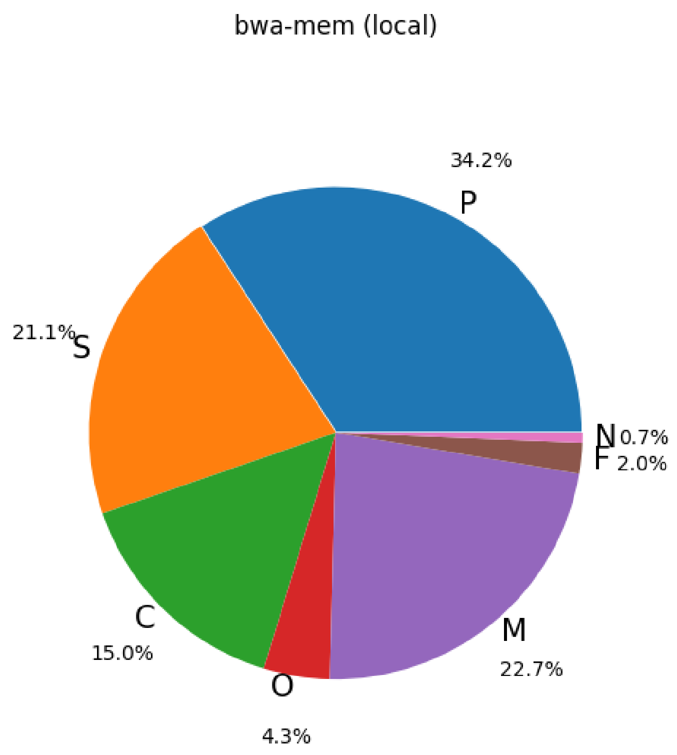

Fig. S3 Alignment percentages for the different classes of reads as assembly quality metric – BWA mem. SQUAT with bwa-mem and percentage of genome assembly mapped reads. P – perfectly matched reads, S – reads with substantial error, C – reads containing clips, O – reads with other errors, M – multi mapped reads, F – failed to map/unmapped reads, N – reads containing N.

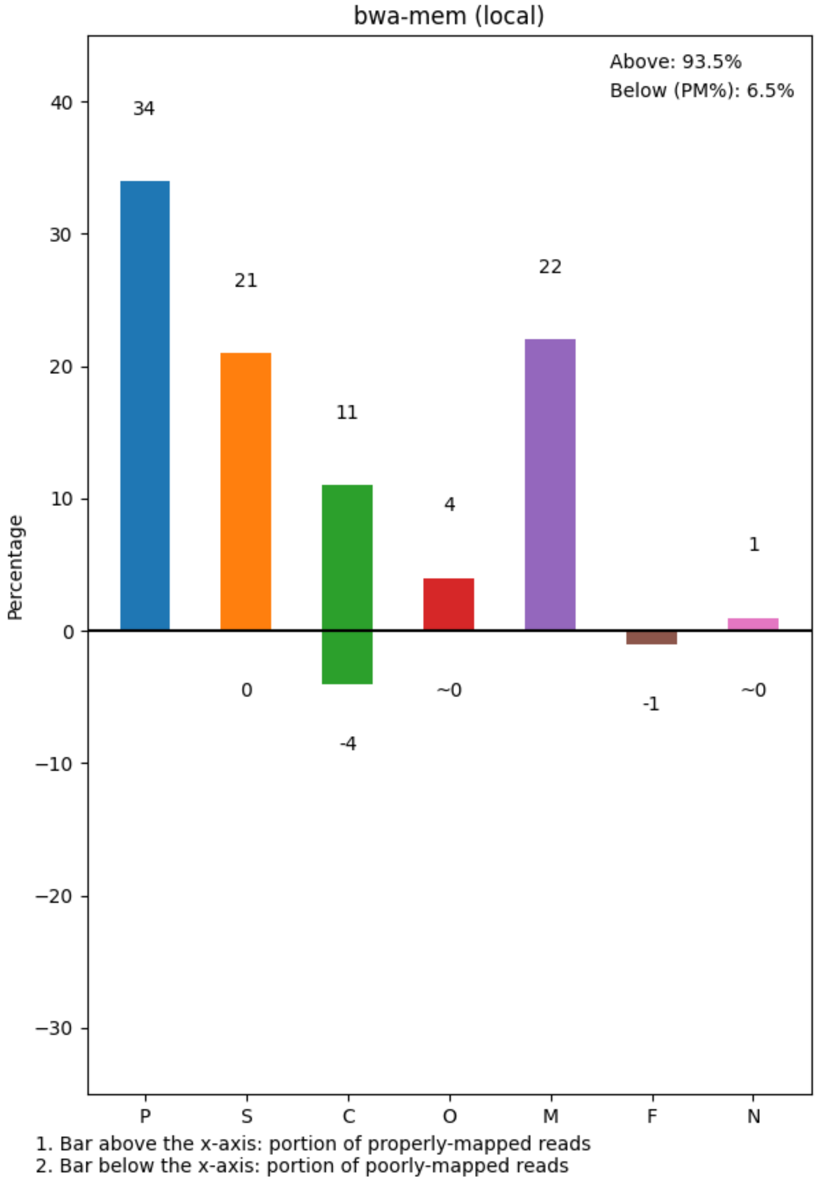

Fig. S4 SQUAT scores for mapped reads. The bar chart uses bars of positive and negative values to represent the percentage of high and poor mapping quality reads accordingly. The sum of the negative values in a bar chart is defined as the poorly-mapped ratio (PM%) of the dataset which defines the overall assembly quality. The dataset passes the assessment if < 20% according to the software standards.

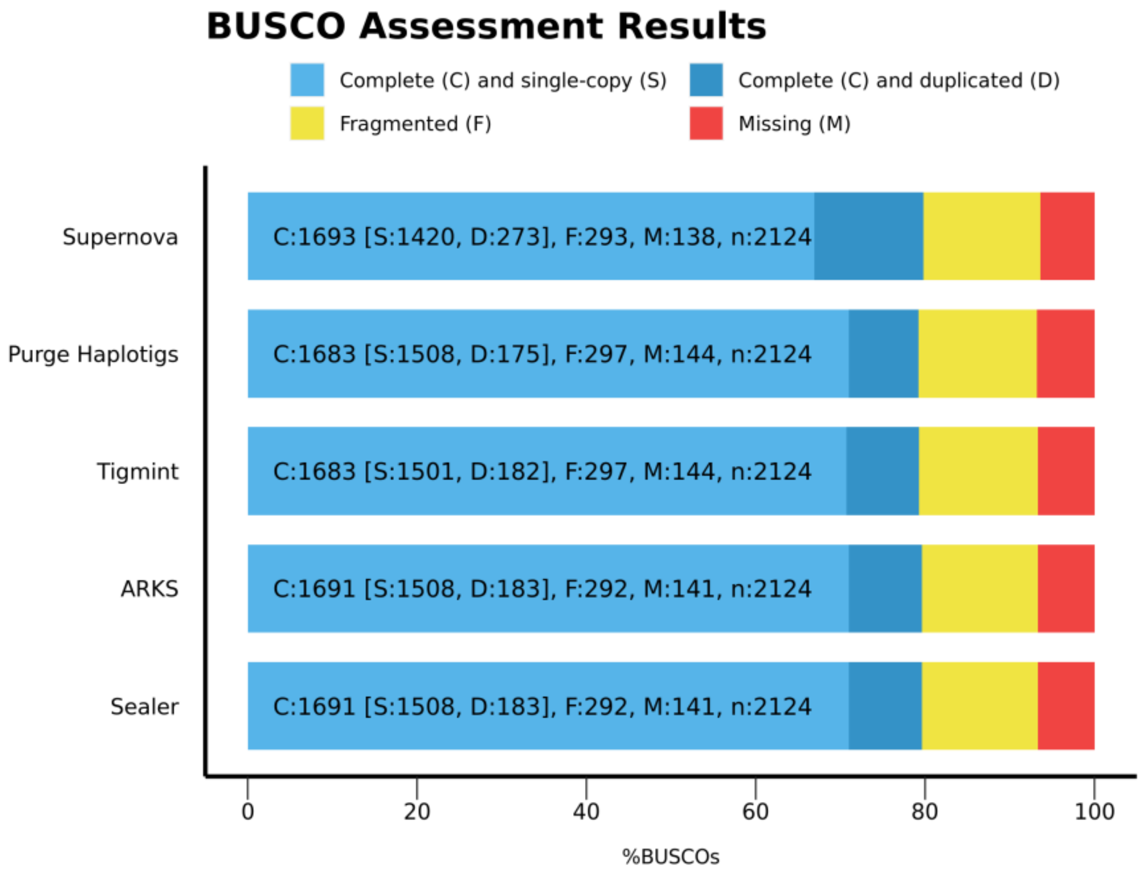

Fig. S5 BUSCO assessment results for each stage of the genome assembly – from top to bottom. The plot reports the percentage of "(C)omplete – (S)ingle copy", "Complete (D)uplicated, "(F)ragmented" and "(M)issing" Endopterygota core gene set for BUSCO v5.2.1 odb10

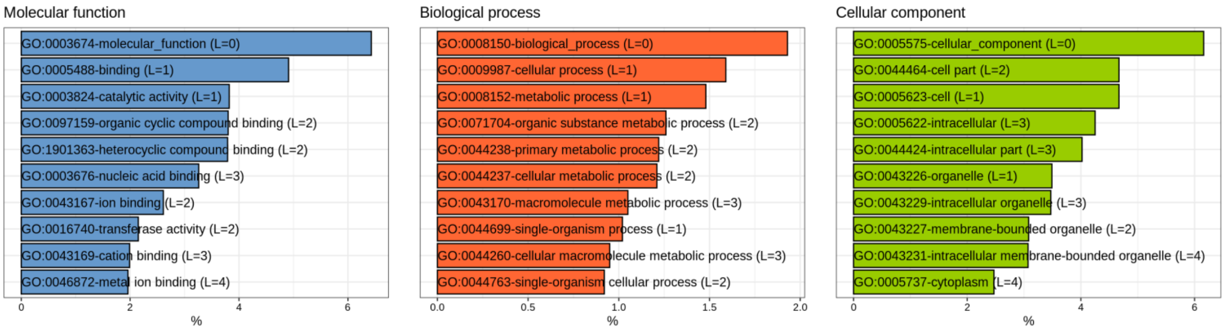

Fig. S6 Frequency of the Gene Ontology (GO) terms of specific level (L), identified in the high quality annotated genes. A total of 18,106 sequences are assigned with a GO term. L0 contains all the GO levels.

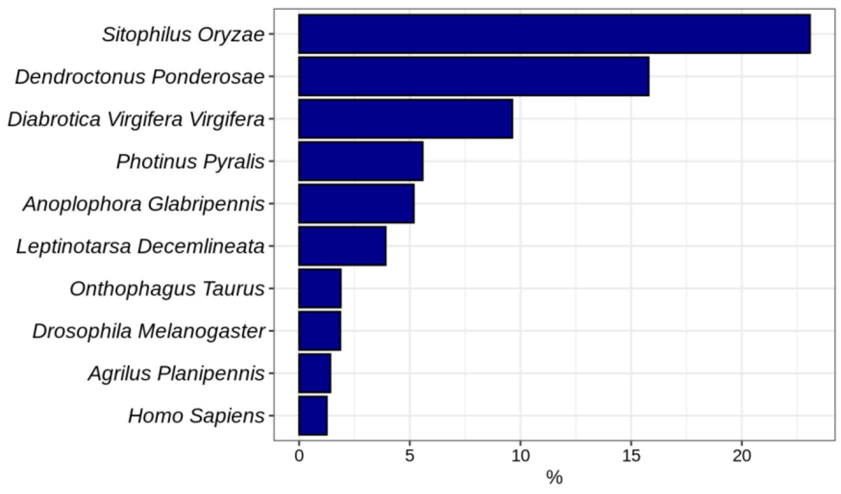

Fig. S7 Top ten species that were hit by alignment within the searched databases, RefSeq and SwissProt/TrEMBL, for the high confidence gene set. The most common species aligning with the annotated genes *is S. Oryzae and D. ponderosae*, both belonging to the *Curculionidae* subfamily *as P. strobi*.

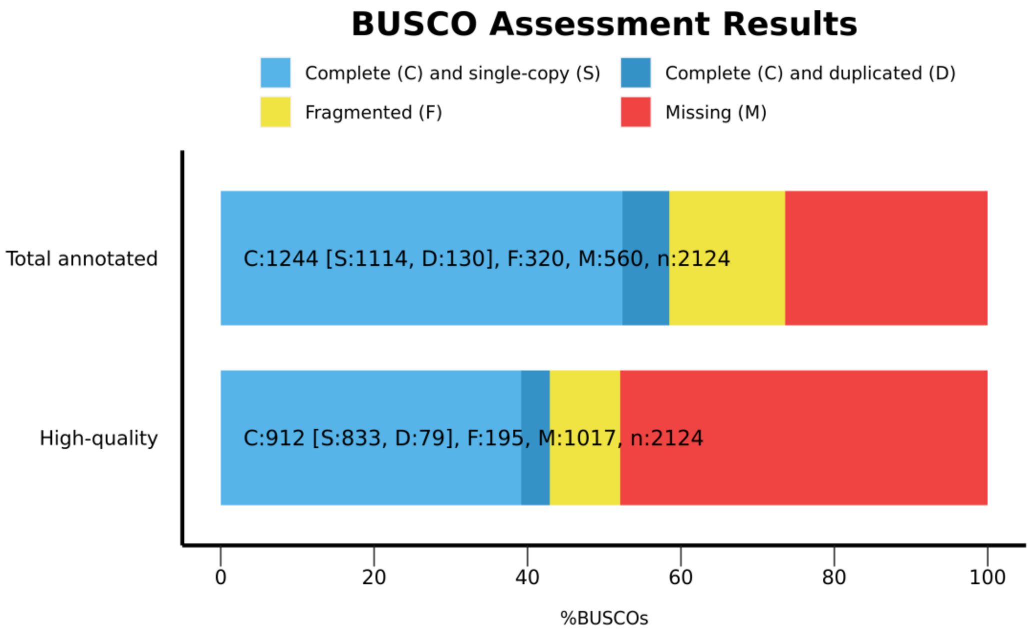

Fig. S8 BUSCO assessment results for the genome annotations – total annotated proteins and high-quality. The plot reports the percentage of "(C)omplete – (S)ingle copy", "Complete (D)uplicated, "(F)ragmented" and "(M)issing" Endopterygota core gene set and odb10

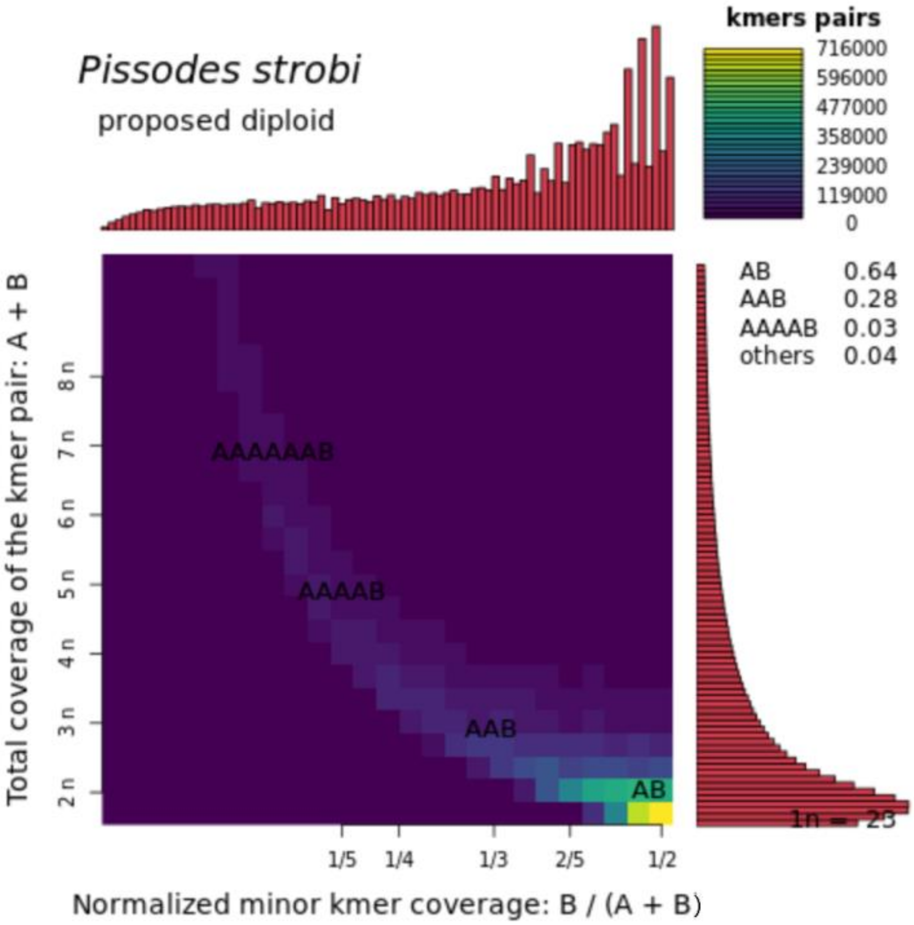

Fig. S9 Smudge plot profiles for heterozygous k-mers *in P. strobi*. The dominant "smudge" is heterozygous diploid (AB) in 64% of the k-mers pairs.

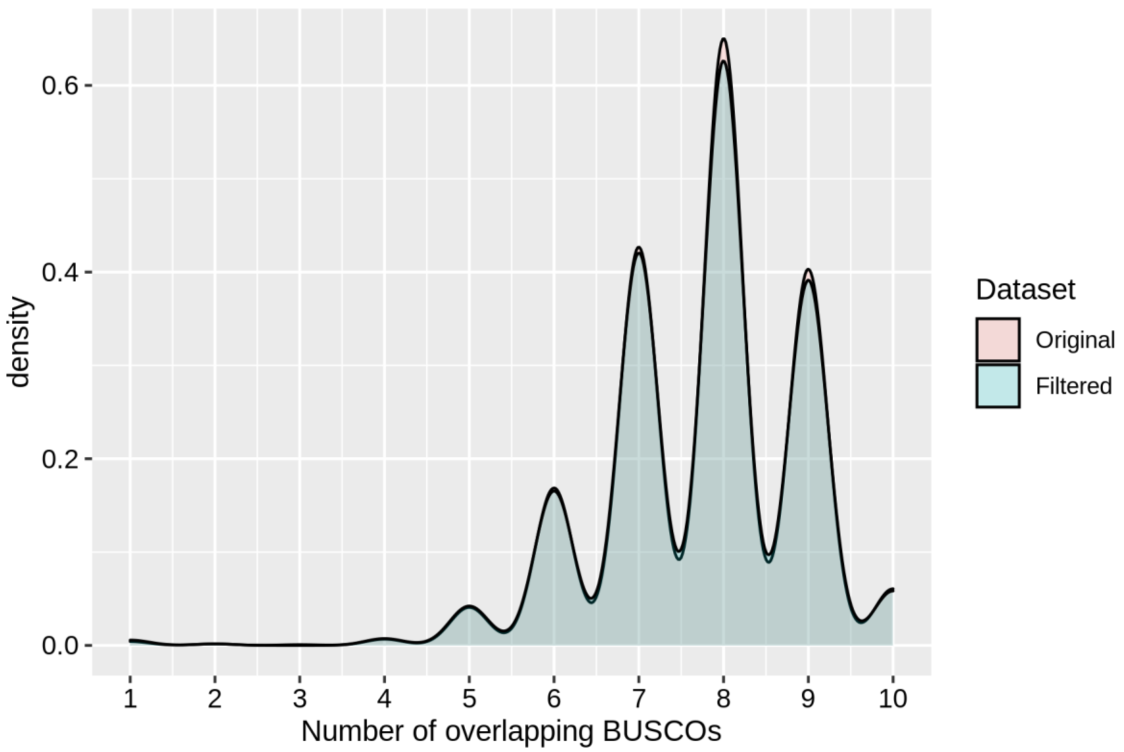

Fig. S10 Number of overlapping BUSCOs among the 10 species used in the phylogenomics analysis, for the total annotated genes (Original) and after fragmentary genes filtering (Filtered). X axis – number of overlapping BUSCOs, Y axis – fraction of the genes annotated in the 10 species. The number of genes annotated in at least one species is 2,122. The total Endopterygota dataset is compounded by 2,124 genes. The filtering removes sequences with sequence gaps in more than 67% of the sequence sites in the Multiple Sequence Alignment.

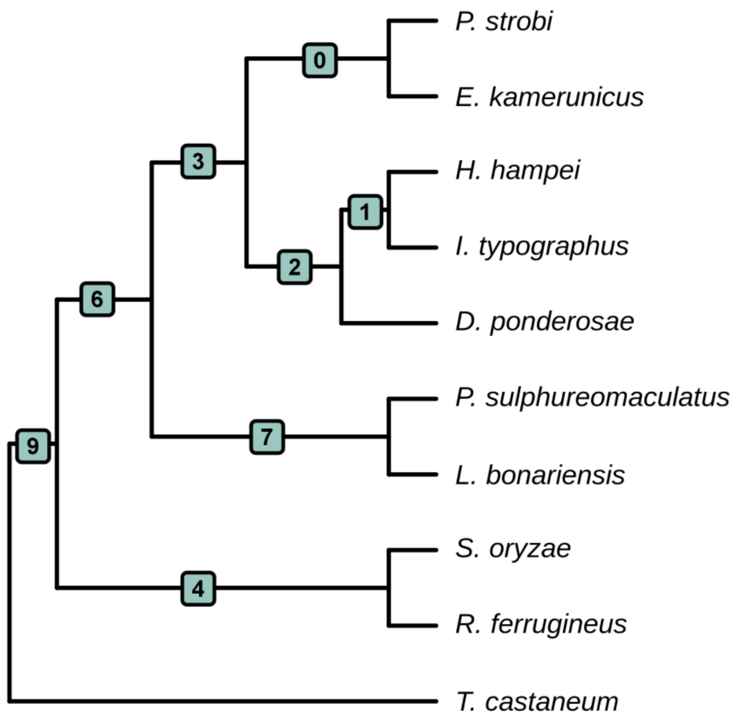

Fig. S11 Nodes numbering used for *phyparts* and DiscoVista (Fig. S12 and S13)

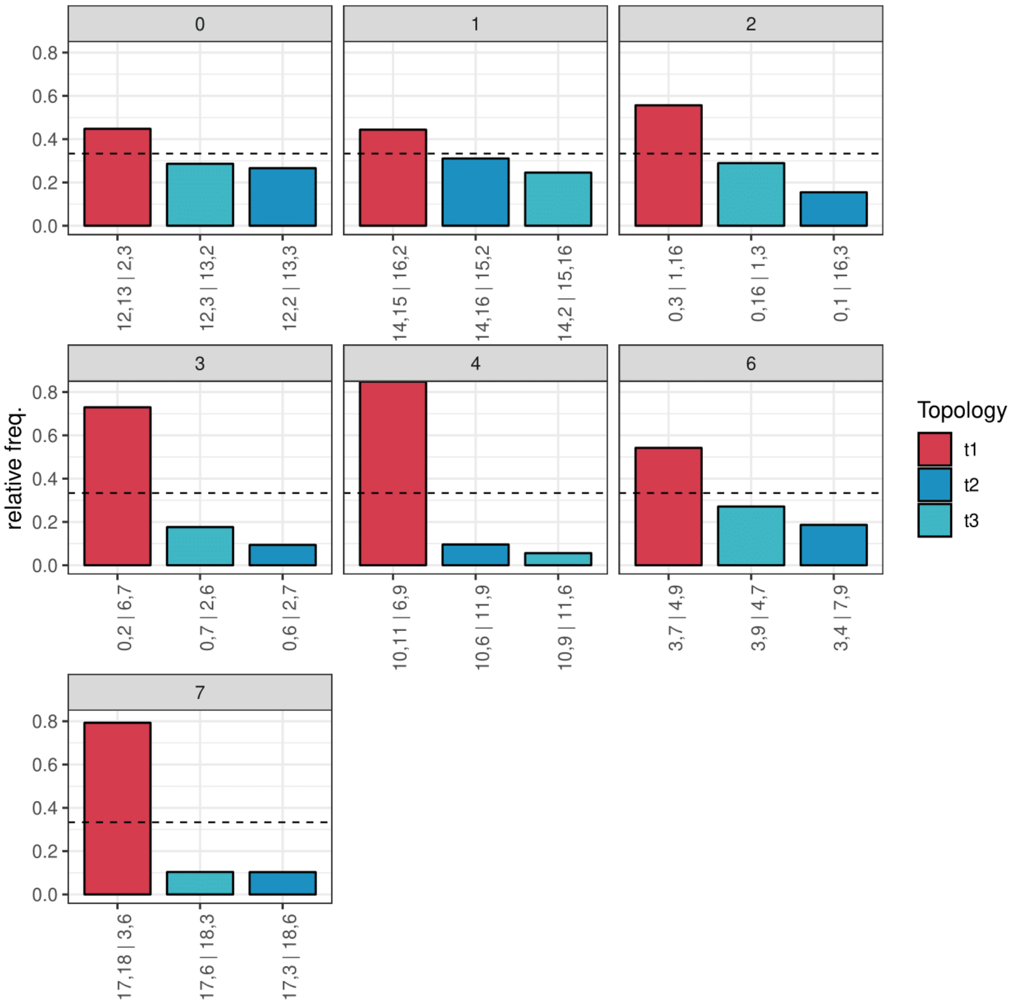

Fig. S12 Quartets relative frequency analysis from DiscoVista. Each panel shows the relative frequency for the internal nodes labeled on the species tree in S11. The bars display the frequency of the main selected quartet (t1 – red) and the alternative quartet topologies (t2 and t3 - blue). A tree topology is assigned when the relative frequency is more than 33% of all the possible topologies (dashed line in the figure).

Node	Concordant	Discordant
0	187	446
1	692	1171
2	869	1106
3	961	814
4	307	71
6	1001	1044
7	1060	445
9 – Out	2009	0

Fig S13 Number of genes supporting each topology node. The values are calculated with *phyparts*, showing the supporting scores for the internal nodes in Fig. S11

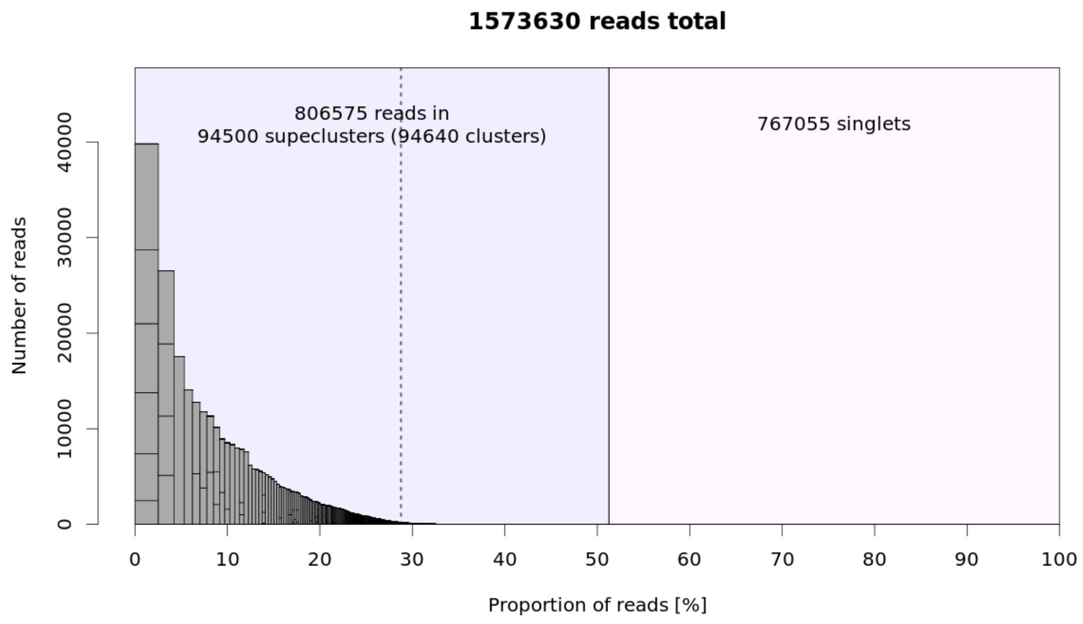

Fig. S14 Repeat composition of clusters generated by RepeatExplorer. X-axis: cumulative proportion of clusters of the genome. Y-axis: numbers of reads. A total set of 1,573,630 reads were selected by the software and 806,575 were arranged in 84,640 clusters. The genomic proportion of these identified repeats was about 52%.

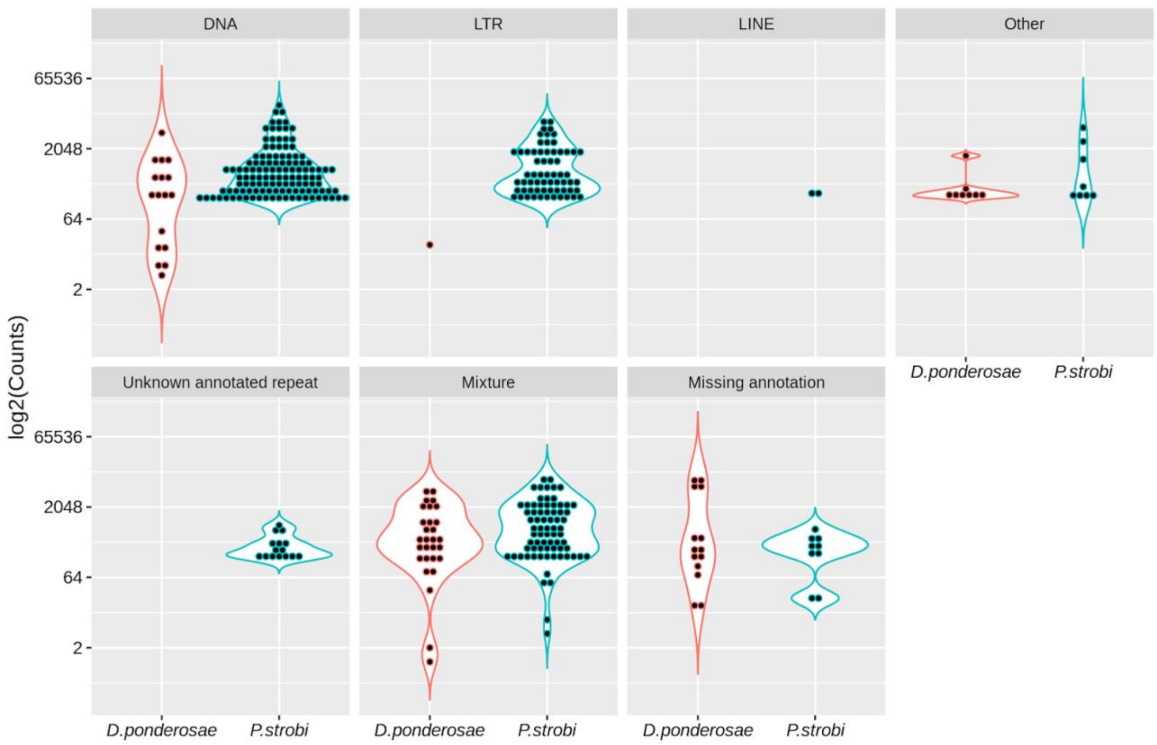

Fig. S15 Comparative analysis *P. strobi* and *D. ponderosae* read clusters. The plot shows the reads counts for each of the highly expressed clusters (n335). The classification of each repeats class was performed through the TEREAN database for the satellite repeats and with the repeats library generated by EDTA.

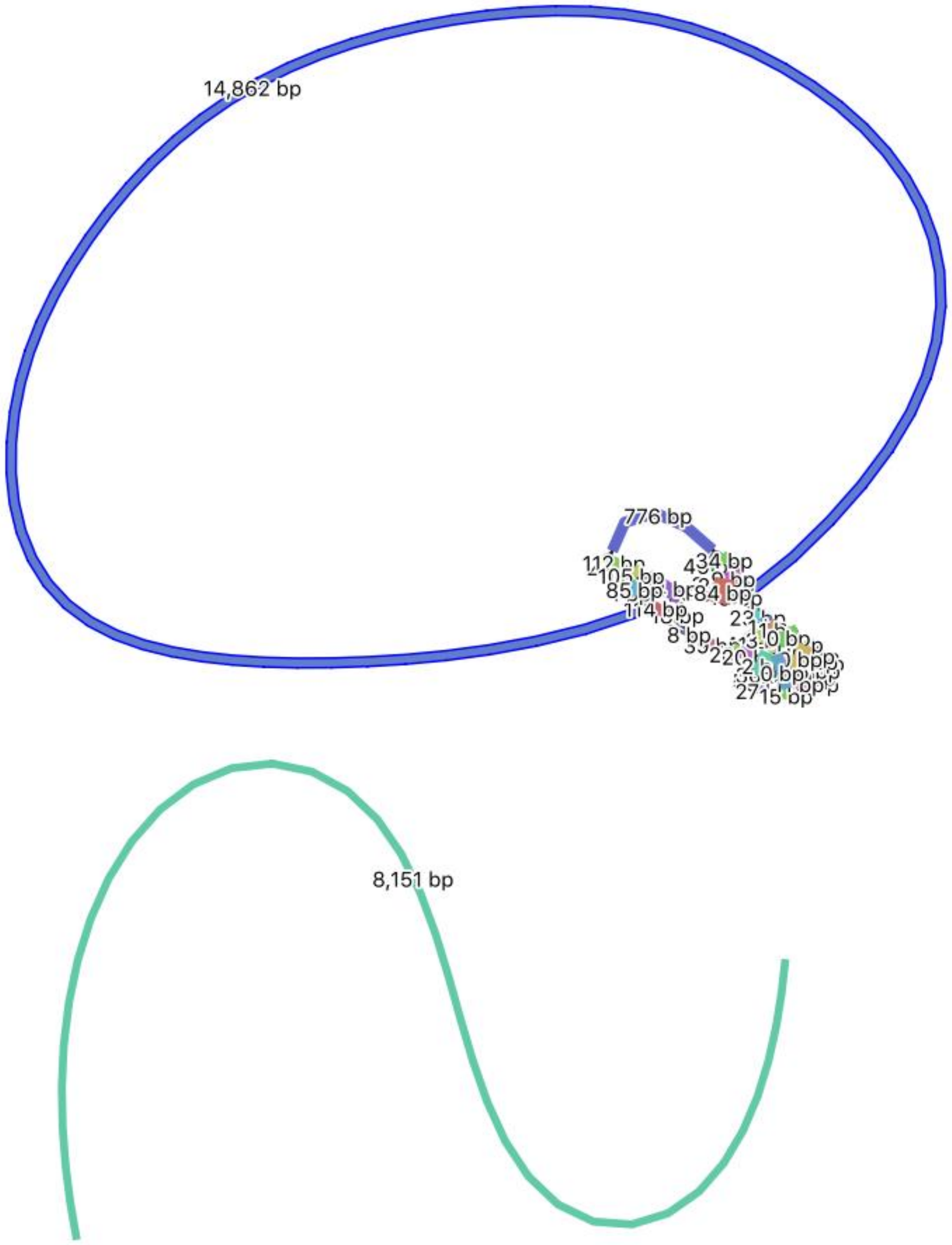

Fig. S16 Bandage graph visualizations of Unicycler assemblies at 62-fold coverage with 10,000 read pairs.

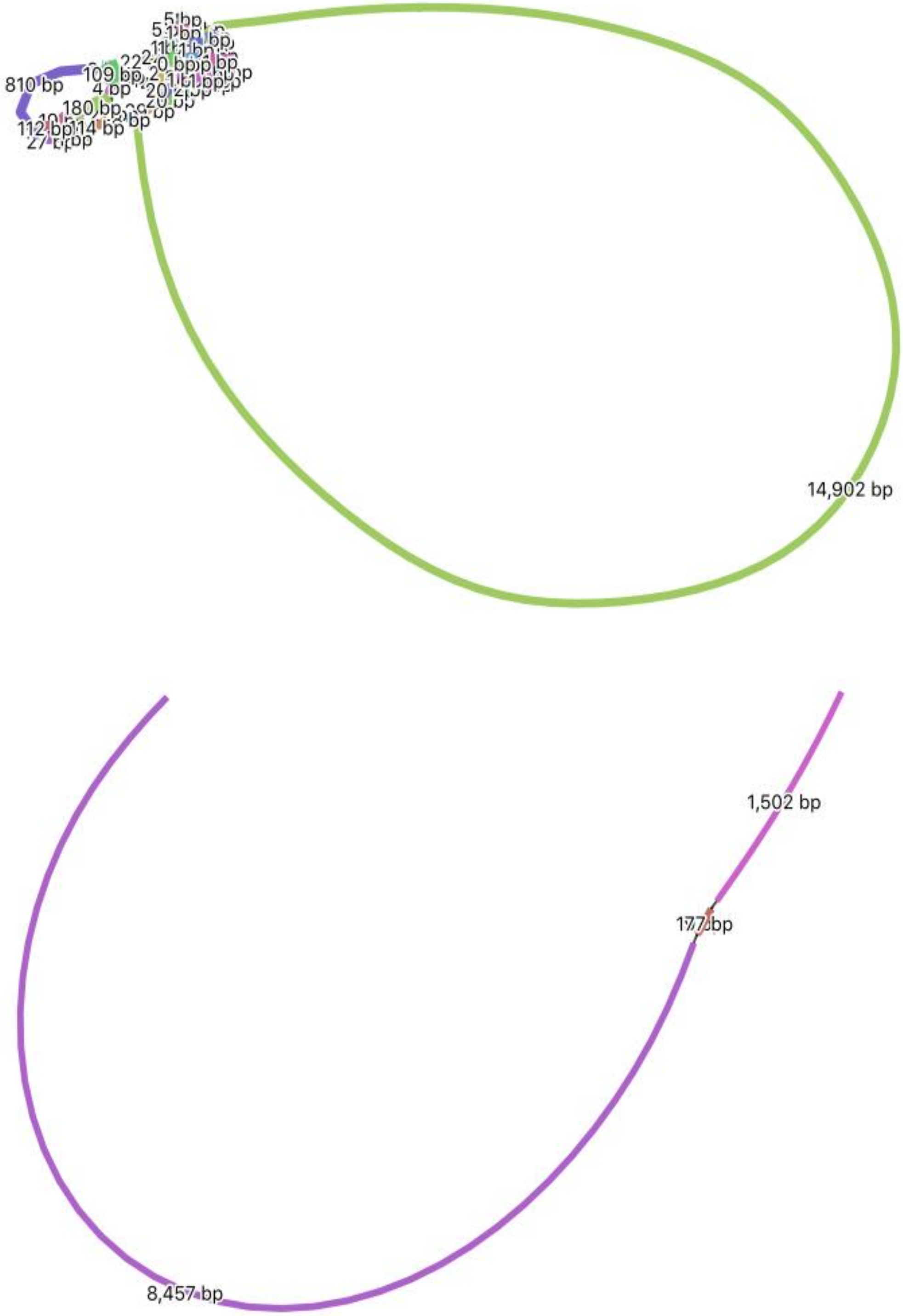

Fig. S17 Bandage graph visualizations of Unicycler assemblies at 96-fold coverage with 15,000 read pairs.

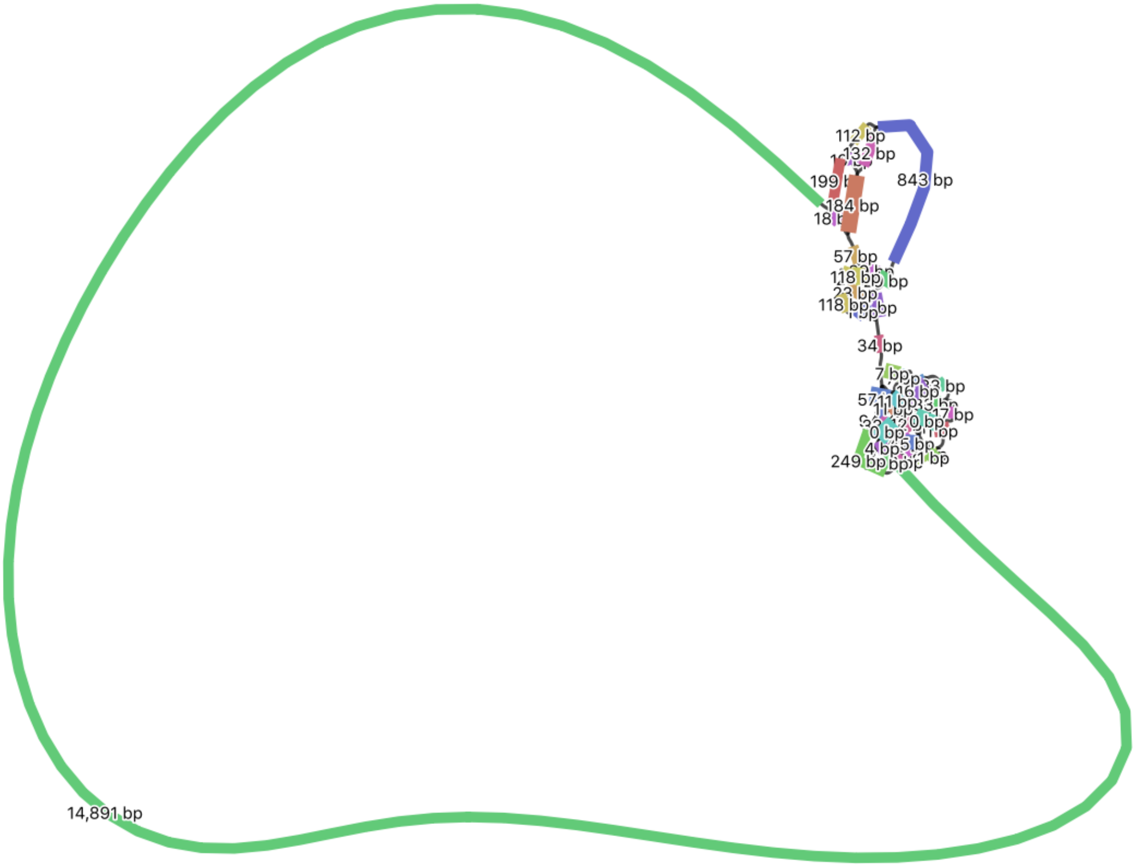

Fig. S18 - Bandage graph visualizations of Unicycler assemblies at 128-fold coverage with 20,000 read pairs.
