# Supplementary material for: The genome of the forest insect pest Pissodes strobi reveals genome expansion and evidence of a Wolbachia endosymbiont
Source: G3 (Bethesda). 2022 Feb 16;12(4):jkac038. doi: 10.1093/g3journal/jkac038 (PMC8982425; doi:10.1093/g3journal/jkac038)
Supplement: jkac038_Table_S2 [file jkac038_table_s2.pdf]

Supplementary Table S2

Table S2 Genome assembly: supernova output statistics at maximum reads sub-sampling. Supernova was run with different number of input reads, selected by --*maxdepth.* The statistics below is generated by Supernova. The complete set of reads with effective coverage of 53.23x was chosen because having the highest N50 Scaffold value

Nº of reads	Effective Coverage	Long scaffolds Kbp	Contig N50 Kb	Scaffold N50 Kb	Assembly size Gb
300 M	21.69	23.83	18.54	30.61	0.62
400 M	27.92	35.44	22.38	51.18	1.17
500 M	34.15	37.78	25.32	68.02	1.45
600 M	39.3	39.06	26.06	75.77	1.61
700 M	45.98	40.32	27.14	80.80	1.71
**831.92 M**	**53.23**	**41.34**	**27.24**	**82.92**	**1.78**
