# Supplementary material for: The genome of the forest insect pest Pissodes strobi reveals genome expansion and evidence of a Wolbachia endosymbiont
Source: G3 (Bethesda). 2022 Feb 16;12(4):jkac038. doi: 10.1093/g3journal/jkac038 (PMC8982425; doi:10.1093/g3journal/jkac038)
Supplement: jkac038_Table_S3 [file jkac038_table_s3.pdf]

Supplementary Table S3

Table S3 Identification and annotation of protein coding genes: gene annotation resources used as evidence in MAKER2. cDNA, transcriptome shotgun assembly (TSA) and RefSeq transcripts are used as *alt_est* evidence in the MAKER2 pipeline (with exception to *P. strobi* that is used as *est* evidence). Proteins downloaded from SwissProt from the *Drosophila* genus are used as *protein* evidence.

Supporting evidence	Species	Access ID	Number of sequences
cDNA	*P. strobi*	GT285068.1 - GT296156.1, KC464331.1, U63328.1	11,091
cDNA	*A. glabripennis*	DQ067275.1, DQ067276.1, AY185203.1, DR108748.1 - DR109303.1, EF583868.1 - EF583870.1, KX660670.1 - KX890113.1, KY039580.1, KY062564.1 - KY287666.1	596
cDNA	*D. ponderosae*	BT126413.1 - BT128693.1, EZ114957.1 - EZ116155.1, GO484341.1 - GO495894.1, GT316901.1 - GT492003.1, JQ855638.1 - JQ855707.1, KC113410.1, KC113439.1, KP736107.1 - KP736166.1, KF444677.1	189,078
cDNA	*S. tribolium*	CN612420.1 - CN612421.1	2
cDNA	*T. castaneum*	AB360761.1 - AB918727.1, AF017415.1 – AF506022.1, AJ005083.1 - AJ973445.1, AM269505.1 - AM922512.1, AY008296.1 - AY887136.1, BK005734.1 - BK008731.1, BN001258.1, CB334789.1 - CB337245.1, CF968201.1 - CF968207.1, CN779602.1, CO049327.1 -CO049345.1, DN643532.1 - DN652253.1, DQ054783.1, DQ060238.1, DQ138189.1 - DQ855506.1, DR753940.1 - DR753993.1, DT769880.1 - DT805528.1, EB748715.1 - EB754265.1, EC009091.1 - EC011169.1, EF117815.1 - EF688530.1, ES544600.1 - ES554556.1, EU008544.1 - EU937812.1, EX149741.1 - EX149815.1, FJ158649.1 - FJ917289.1, FM163173.1, FN295953.1 - FN824497.1, GQ202020.1 - GQ368184.1, GU111762.1 - GU727869.1, HE608844.1, HM234671.1 - HM622134.1, HQ110094.1 - HQ824707.1, JF682841.1 - JQ922422.1, JX099777.1 - JX569831.1, KC161573.1 - KC688266.1, KF192693.1 - KF951599.1, KJ405472.1 - KJ500311.1, KM216386.1 - KM925014.1, KP120763.1 - KP843191.1, KT778599.1, KX553973.1 - KX812753.1, KY368366.1 - KY971527.1, LC154964.1 - LC191269.1, LS991960.1 - LS991974.1, LT908025.1 - LT908033.1, MF467204.1 - MF467212.1, MG011448.1 - MG913606.1, MH664125.1 - MH664127.1, Z69735.1 - Z69743.1	65,269
TSA	*P. strobi*	GAEO01000001.1 - GAEO01004939.1	4,940
TSA	*D. ponderosae*	GABX01000001.1 - GABX01000059.1	60
TSA	*D. ponderosae*	SRR1702878 - SRR1703019	197,866
RefSeq transcripts	*Endopterygota Taxon id – 33392*	XM; NM	1,389,102
SwissProt proteins	*Drosophila genus*	DROME, DROPS, DROVI, DROYA, DROSI, DROER, DROSE, DROAN, DROPE, DROGR (top most frequent species)	5,915
