# Supplementary material for: The genome of the forest insect pest Pissodes strobi reveals genome expansion and evidence of a Wolbachia endosymbiont
Source: G3 (Bethesda). 2022 Feb 16;12(4):jkac038. doi: 10.1093/g3journal/jkac038 (PMC8982425; doi:10.1093/g3journal/jkac038)
Supplement: jkac038_Table_S4 [file jkac038_table_s4.pdf]

Supplementary Table S4

Table S4 Identification and annotation of protein coding genes: list of SRA reads and pools used by Rnabloom for the *D. ponderosae* TSA. Rnabloom is run with the following command: *Java –jar RNA-Bloom.jar -ntcard -fpr 0.005 -k 25 -t 48 -pool ReadsList.txt -revcomp-right -name Mpb -outdir Mpb*, where ReadsList.txt reflects the read pools listed in the table.

D. ponderosae reads pool	SRA reads
FAFB	SRR170287, SRR170290, SRR170291, SRR170294
FAMG	SRR1702898, SRR1702901, SRR1702904, SRR1702910
FJFB	SRR1702913, SRR1702916, SRR1702919, SRR1702923
FJMG	SRR1702925, SRR1702927, SRR1702929, SRR1702930
MAFB	SRR1702932, SRR1702933, SRR1702934, SRR1702950
MAMG	SRR1702966, SRR1702979, SRR1702987, SRR1702988
MJFB	SRR1702992, SRR1703009, SRR1703010, SRR1703012
MJMG	SRR1703014, SRR1703016, SRR1703018, SRR1703019
