# Supplementary material for: The genome of the forest insect pest Pissodes strobi reveals genome expansion and evidence of a Wolbachia endosymbiont
Source: G3 (Bethesda). 2022 Feb 16;12(4):jkac038. doi: 10.1093/g3journal/jkac038 (PMC8982425; doi:10.1093/g3journal/jkac038)
Supplement: jkac038_Table_S5 [file jkac038_table_s5.pdf]

Supplementary Table S5

Table S5 Phylogenomic comparisons: representative genomes from GenBank used for BUSCO annotation and phylogenomics.

Species	GenBank access	Version
Tribolium castaneum	GCA_000002335	5.2
Rhynchophorus ferrugineus	GCA_014490705	1.0
Sitophilus oryzae	GCA_002938485	2.0
Listronotus bonariensis	GCA_014170235	1.0
Pachyrhynchus sulphureomaculatus	GCA_019049505	1.0
Dendroctonus ponderosae	GCA_000355655	1.0
Ips typographus	GCA_016097725	1.0
Hypothenemus hampei	GCA_001012855	1.1
Elaeidobius kamerunicus	GCA_014849505	1.0
