# Supplementary material for: The genome of the forest insect pest Pissodes strobi reveals genome expansion and evidence of a Wolbachia endosymbiont
Source: G3 (Bethesda). 2022 Feb 16;12(4):jkac038. doi: 10.1093/g3journal/jkac038 (PMC8982425; doi:10.1093/g3journal/jkac038)
Supplement: jkac038_Table_S6 [file jkac038_table_s6.pdf]

Supplementary Table S6

Table S6 *Wolbachia* spp. assembly statistics and reads sub-sampling. ABySS was run with different number of input reads and different k values. The summary statistics is shown for the k value which gives the highest contiguity per sub-sampled genome coverage. The maximum number of reads with effective coverage of 375-fold was chosen (highlighted) because it produced the highest N50 value and the highest reconstructed genomes size.

Coverage	Abyss k	Number of scaffolds	N50 (bp)	Reconstructed genome size (bp)
**375**	**68**	**247**	**6,940**	**1,192,984**
300	48	398	2,325	835,816
200	48	282	1,483	420,495
100	84	9	2,568	18,326
50	60	7	3,833	14,744
