# Supplementary material for: The genome of the forest insect pest Pissodes strobi reveals genome expansion and evidence of a Wolbachia endosymbiont
Source: G3 (Bethesda). 2022 Feb 16;12(4):jkac038. doi: 10.1093/g3journal/jkac038 (PMC8982425; doi:10.1093/g3journal/jkac038)
Supplement: jkac038_Table_S8 [file jkac038_table_s8.pdf]

Supplementary Table S8

Table S8 GenomeScope2.0 estimates for *P. strobi*. The values calculated by the k-mers profiles are the genome length, % heterozygosity and % unique sequences; the last column shows the average of the k-mers 21-29.

P. strobi	21	23	25	27	29	**Avg.**
Haploid genome length (Gbp)	1.758	1.762	1.762	1.767	1.762	**1.762**
Heterozygous (%)	2.74	2.65	2.57	2.47	2.4	**2.56**
Unique sequences (%)	43.4	45.9	47.8	49.6	51.1	**47.56**
