# Supplementary material for: The genome of the forest insect pest Pissodes strobi reveals genome expansion and evidence of a Wolbachia endosymbiont
Source: G3 (Bethesda). 2022 Feb 16;12(4):jkac038. doi: 10.1093/g3journal/jkac038 (PMC8982425; doi:10.1093/g3journal/jkac038)
Supplement: jkac038_Table_S9 [file jkac038_table_s9.pdf]

Supplementary Table S9

Table S9 Summary of all annotated repeats in the *P. strobi* and *D. ponderosae* genomes. The main repeat classes highlighted in bold. Each class is further subdivided in subclasses. The quantification is shown as counts, number of masked base pairs and percent masked genome.

Class	Count	Bp Masked	% masked
**DIRS**	1448	554665	0.03
**DNA**			
DTA	835839	279357753	15.24
DTC	226398	60607971	3.31
DTH	74173	19517433	1.06
DTM	534390	170266118	9.29
DTT	29466	7428204	0.41
Helitron	152740	43961937	2.4
**Helitron**	208	109413	0.01
**LINE**			
Unknown	54023	23934114	1.31
**LTR**			
Copia	46975	19196968	1.05
Gypsy	307419	157183152	8.58
Unknown	309153	112595223	6.14
**MITE**			
DTA	14111	2779365	0.15
DTC	7927	1475120	0.08
DTH	7309	1299602	0.07
DTM	57906	10160357	0.55
DTT	534	74181	0
**Maverick**	1093	672740	0.04
**Penelope**	9908	3262958	0.18
**TIR**			
Kolobok	758	434890	0.02
Novosib	97	77827	0
PIF_Harbinger	567	251874	0.01
PiggyBac	1330	432817	0.02
Tc1_Mariner	6256	1569364	0.09
**Unknown**	235060	63079143	3.44
**Mixture**	2235	917023	0.05
Total ***P. strobi***	2917323	981200212	**53.53%**

Class	Count	Bp Masked	% masked
**DNA**			
DTA	10262	2182169	1.08
DTC	15205	3015590	1.49
DTH	4129	831309	0.41
DTM	24634	5549959	2.75
DTT	2161	400460	0.20
Helitron	16470	3333789	1.65
**LTR**			
Copia	23	7180	0.00
Unknown	6364	1346226	0.67
**MITE**			
DTA	1946	352337	0.17
DTC	353	73131	0.04
DTH	315	60310	0.03
DTM	1804	297687	0.15
DTT	4	448	0.00
Total ***D. ponderosae***	83670	17450595	**8.65%**
